# Three-Dimensional Reconstruction of the Bony Nasolacrimal Canal by Automated Segmentation of Computed Tomography Images

**DOI:** 10.1371/journal.pone.0155436

**Published:** 2016-05-17

**Authors:** Lucia Jañez-Garcia, Federico Saenz-Frances, Jose M. Ramirez-Sebastian, Nicolas Toledano-Fernandez, Maria Urbasos-Pascual, Luis Jañez-Escalada

**Affiliations:** 1 Ramon Castroviejo Institute of Ophthalmological Investigations, Complutense University, Madrid, Spain; 2 Ophthalmology Department, Clinico Universitario San Carlos Hospital, Complutense University, Madrid, Spain; 3 Universitario de Fuenlabrada Hospital, Madrid, Spain; 4 Institute of Knowledge Technology, Complutense University, Madrid, Spain; University of Palermo, ITALY

## Abstract

**Objective:**

To apply a fully automated method to quantify the 3D structure of the bony nasolacrimal canal (NLC) from CT scans whereby the size and main morphometric characteristics of the canal can be determined.

**Design:**

Cross-sectional study.

**Subjects:**

36 eyes of 18 healthy individuals.

**Methods:**

Using software designed to detect the boundaries of the NLC on CT images, 36 NLC reconstructions were prepared. These reconstructions were then used to calculate NLC volume. The NLC axis in each case was determined according to a polygonal model and to 2^nd^, 3^rd^ and 4^th^ degree polynomials. From these models, NLC sectional areas and length were determined. For each variable, descriptive statistics and normality tests (Kolmogorov-Smirnov and Shapiro-Wilk) were established.

**Main Outcome Measures:**

Time for segmentation, NLC volume, axis, sectional areas and length.

**Results:**

Mean processing time was around 30 seconds for segmenting each canal. All the variables generated were normally distributed. Measurements obtained using the four models polygonal, 2^nd^, 3^rd^ and 4^th^ degree polynomial, respectively, were: mean canal length 14.74, 14.3, 14.80, and 15.03 mm; mean sectional area 15.15, 11.77, 11.43, and 11.56 mm^2^; minimum sectional area 8.69, 7.62, 7.40, and 7.19 mm^2^; and mean depth of minimum sectional area (craniocaudal) 7.85, 7.71, 8.19, and 8.08 mm.

**Conclusion:**

The method proposed automatically reconstructs the NLC on CT scans. Using these reconstructions, morphometric measurements can be calculated from NLC axis estimates based on polygonal and 2^nd^, 3^rd^ and 4^th^ polynomial models.

## Introduction

The nasolacrimal drainage system, or tear duct, is mostly lodged in the nasolacrimal canal (NLC). The bony NLC is defined, laterally, by the lacrimal groove of the medial side of the maxilla and by the lacrimal hook of the lacrimal bone, which scrolls over the outer edge of the canal's upper orifice; and medially, by the lacrimal bone superiorly and by the lacrimal process of the inferior nasal concha inferiorly. The medial canal wall is comprised of the lacrimal bone in its upper region and by the lacrimal process of the inferior nasal concha in its lower portion. Obstruction of the tear duct may cause epiphora and dacryocystitis [1–9] and has been associated with a greater risk of endophthalmitis following intraocular surgery [10, 11]. The most frequent causes of secondary acquired obstruction are facial surgery, trauma, neoplasm, sarcoidosis, and Wegener granulomatosis [1–9]. In contrast, primary acquired obstruction is idiopathic and involves gradual chronic inflammation and fibrosis [1–9]. Anatomical characteristics inherent to skull dimensions may also be a cause of obstruction [1–9]. Few studies have examined the anatomy of the lacrimal system, and most such studies have been based on visual inspection and manual analysis of computed tomography (CT) scans, a procedure that can be inaccurate and time consuming [1–9].

Detailed knowledge of the anatomy of the NLC would provide useful information for diagnostic purposes and for planning interventions for tear duct obstruction. Herein, we describe a new completely automated method of bony NLC reconstruction whereby segmentation software is used to determine the canal's size and main morphometric characteristics in high-resolution CT scans.

## Materials and Methods

We retrieved the electronic medical records of patients that included a DICOM computerized tomography (CT) of the nasolacrimal canal (NLC) performed from January 2008 to December 2014 at the Hospital Universitario de Fuenlabrada (Madrid, Spain). All patients under 18 years of age were excluded. The study protocol was approved by our institution’s Review Board (Ethics Committee of The Hospital Clínico Universitario San Carlos of Madrid, Spain and complied with the guidelines of the Declaration of Helsinki. Being a retrospective study, informed consent was not required although the study examiners were masked to patients' personal data to protect their confidentiality. All the study participants were Caucasian.

CT scans were required to have a high spatial resolution (pixel spacing ≤ 0.5mm, slice thickness 0.625 mm) and to be reconstructed with standard or bone kernels. Scans showing a sinus condition were not selected.

CT scans obtained in patients who had previously undergone previous tear duct imaging and/or irrigation or those undergoing a scan after dacrocystorhinostomy (DCR) surgery were also excluded. We also excluded scans obtained in patients with infection, inflammation, neoplasm, malformations or any pathology which may involve the NLC. Those undergoing trauma or surgery involving the NLC were also excluded.

CTs were generated using GE LightSpeed16 equipment without contrast and with the subject in supine position. The plane of imaging was perpendicular to the gantry table. A contiguous helical axial data set was obtained from below the maxilla or skull base to above the frontal sinus. Voxel data: pixel spacing 0.292969/0.292969 to 0.417969/0.417969 mm and slice thickness 0.625 mm with neither gaps nor overlap between consecutive slices. Protocol for head/skull-base/petrous bone: 120 kVp, 240 mA fixed value without modulation, slice thickness 0.625 mm with the same image interval, gantry angle 0°, FOV 250 mm, CTDI: 74.02 mGy. CT volumes were exported in DICOM format. Age and sex were also recorded.

A software was developed to virtually reconstruct the bony NLC. From this automatic reconstruction, stereometric parameters of the NLC were obtained as follows.

### Models of the NLC

To provide the formal description and characteristic parameters of each NLC, two types of models have been created, each providing different information about the 3-D structure of the NLC: the axis models and surface models.

#### Axis models

This type of models was derived from the centroids of the NLC regions segmented in each CT slice, by interpolating them or by fitting mathematical functions. Centroids were calculated according to established methods (Gonzalez and Woods, 2002 [12]). The axis model may be described as the sequence of centroids *(c*_*1*_, *c*_*2*_, *…*, *c*_*p*_*)*—*c*_*i*_ having *(x*_*i*,_ *y*_*i*,_ *z*_*i*_*)* as its physical coordinates-, and as any function interpolating them: polygonal, sinc, or other. However, as a result of various factors affecting segmentation, each computed centroid may deviate randomly from its true position. Thus to minimize the impact of those errors, a new type of axis model has been obtained by fitting different smooth functions to the sequence of all centroid points: third and fourth order polynomials were selected because of their smoothness, few parameters and at least one inflexion point; second order polynomial has been added for comparison; thus the family of polynomial models *s(z)* for the canal axis adopted the following parametric expression:

$$s(z)\equiv \left\{ \begin{array}{l} x(z)=a_{0}+a_{1}z+a_{2}z^{2}+a_{3}z^{3}+a_{4}z^{4},\;{}_{}sagital\;{}^{}coordinate\\ y(z)=b_{0}+b_{1}z+b_{2}z^{2}+b_{3}z^{3}+b_{4}z^{4},\;{}_{}coronal\;{}^{}coordinate\\ z\in \left[z_{1}-\frac{\delta }{2},{}^{}z_{p}+\frac{\delta }{2}\right]\subset \mathbb{R},\;{}_{}depth\;{}^{}coordinate \end{array}\right.$$ (1)

where *δ* is the slice spacing given by the difference between the third components of DICOM tags “Image Position (Patient)” (0020,0032) in two contiguous slices, and *z*_1_ and *z*_*p*_ are the *z*-coordinates of centroids of the canal region in the first and last slices of the NLC; coordinates were expressed in mm and the origin of the coordinate system was that of the whole CT (i.e. geometrical centre of the upper left voxel in the first slice of the CT series).

To construct the polynomial model for the NLC axis corresponding to the polynomial function of degree 2, 3 or 4, the coefficients *a*_*i*_ and *b*_*i*_ of its *x(z)* and *y(z)* components were obtained for each NLC by separately fitting *x(z)* and *y(z)* to coordinates *(x*_*i*,_ *z*_*i*_*)* and *(y*_*i*,_ *z*_*i*_*)* (i = 1, 2, …,p) of the NLC centroids, respectively, by the least squares method; the square of Pearson correlation coefficients was used to assess the goodness of fit.

#### Surface model

To describe the external surface of the NLC, a numerical model was obtained for each NLC of all subjects by 3-D interpolating and smoothing the contours of the automatically segmented NLC sections, followed by rendering of peripheral isosurface.

### Length of the NLC

**Fig 1:**
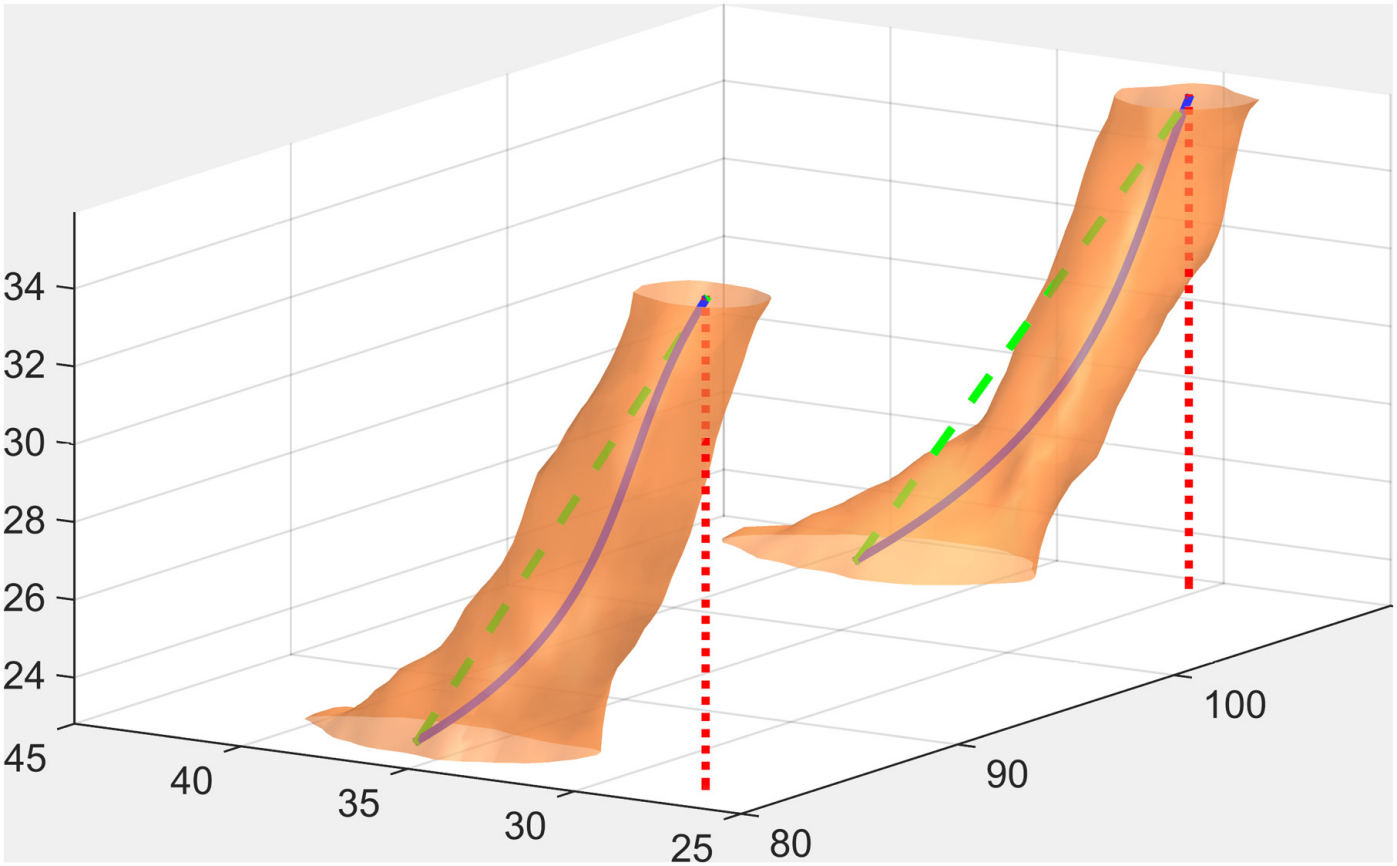
Different evaluations of NLC length: axial (dotted red line), end-to-end (dashed green line) and true (continuous blue line).

**Fig 2:**
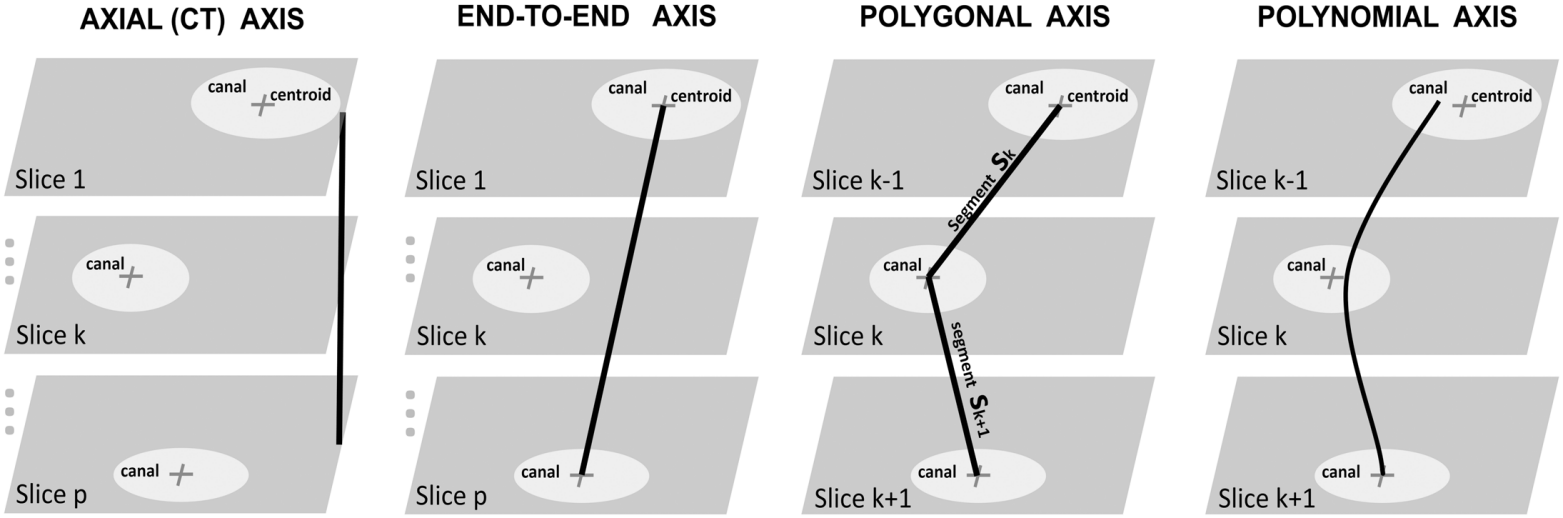
Depiction of the four methods to compute NLC length: axial, end-to-end, polygonal, polynomial.

The length of the 3-D curve described by each axis model provided a new method of measuring NLC length; thus polygonal length and three polynomial lengths were compared with the traditional methods based on axial distance and end-to-end distance (Figs 1 and 2).

### Cross-sectional area

The area of the canal section as it appears on the CT image (axial area) has been frequently calculated with many different computer aids but based on points or contours that are identified visually and marked manually. However, this manual procedure is cumbersome determining that measurements are usually only taken at one or few NLC levels; also in many cases rather than measuring the irregular shape of the canal region, the area measured is an ellipse or other regular shape that is manually fitted to the region or otherwise only estimated from anterior-posterior and transverse diameters (e.g. Lee et al. [13]).

The new method proposed here to calculate cross-sectional NLC areas in axial slices is a fully automated three-step computer procedure: in the first step, the canal is segmented from the rest of the image; in the second step, the program counts the number of pixels in the region identified; and in the final step, this number is multiplied by the area covered by an individual pixel, obtained by multiplying the two components of the “pixel spacing tag” (0028,0030) provided in the DICOM image header. However automation improves speed and objectivity but is not sufficient, because the sectional area obtained is overestimated unless the CT slice is perpendicular to the canal, as in the manual method. Two different approaches have been developed to get the sectional area orthogonal to the canal: estimation from the axial area and direct calculation.

Direct calculation method is based on the now available models of NLC axis, which can provide the canal’s direction at any point *k* of its track and easily identify the plane $P_{k}^{⊥}$ orthogonal to it, which is the sectional plane where the true sectional area $A_{k}^{⊥}$ of the canal must be calculated. An automatic implementation of this calculation was then defined, similar to the visual and manual methods described by Estes et al [9]. This firstly involves computing the geometrical tangent to the curve of the selected axis model at point *k* and the equation of its orthogonal plane $P_{k}^{⊥}$, then generating by image interpolation a new slice reconstructed at $P_{k}^{⊥}$ and finally segmenting the canal and assessing its area to directly obtain the value of $A_{k}^{⊥}$, all as automated steps with our software.

Estimation of the orthogonal area from the axial one is however feasible and faster when it can be assumed that the NLC path doesn’t change abruptly and the slicing orientation is not strongly deviated from orthogonality to NLC at point *k*; then the true sectional area $A_{k}^{⊥}$ can also be obtained as the projection’s area on $P_{k}^{⊥}$ of the axial section, and is given by $A_{k}^{⊥}=A_{k}\text{cos}\beta_{k}$, where *A*_*k*_ is the sectional area of axial slice at the same point and *β*_*k*_ is the angle between canal and CT axes. This equation implies that orthogonal areas are always equal to or less than the corresponding axial areas, as expected.

In this work, four axis models were constructed: polygonal, 2^nd^, 3^rd^ and 4th order polynomials; each model provides its own basis to measure the orthogonal cross-sectional area at all the slicing points of each NLC; so far, five different variables related to sectional area of the NLC have been studied, four orthogonal and one axial, all areas being computed automatically: axial, polygonal, polynomial-2, polynomial-3 and polynomial-4 areas.

### Volume

In its simplest approach, NLC volume has been derived from geometrical solids, such as cylinders, ellipsoids or spheroids, derived from orthogonal radii and other manual measurements or visually fitted to canal images. However as the canal’s shape is irregular, geometric approximations are too inaccurate. To determine the volume of the canal's real shape we used automatic segmentation to identify the voxels making up the NLC in each CT slice.

Before automatically calculating NLC volume, the consistency of data in all relevant DICOM tags needs to be verified, given that some errors have been reported [14, 15].

We thus checked for all slices that the difference between consecutive slices in “slice-location” (0020,1041) equals the difference in the third component of “image-position-patient” (0020,0032). It was also checked that the coordinate system indicated by the direction cosines in “image orientation patient” (0020,0037) was the canonical one.

To compute NLC volume, the volume of each voxel is calculated by simply multiplying its base area by its depth. Base area is the product of the two “pixel spacing” numbers in tag (0028,0030) and depth is the difference in “slice location” (0020,1041) with respect to the next slice. Alternatively, “slice thickness” (0018,0050) can also be used for depth provided there is no gap or overlap between consecutive slices. NLC volume is then given by the sum of volumes of all the voxels that the segmentation software has marked as included in the NLC. If the approach here proposed reveals fruitful, future research will include an additional correction at both ends of the NLC, by adding or subtracting the wedge between the outer surface of the slice now delimiting each end of the canal and its true delimiting plane which is orthogonal to the end of its axis.

The effects on goodness of fit due to polynomial order (2, 3, 4), coordinate (x, y) and NLC laterality (right, left) were evaluated using a factorial design 3x2x2 with repeated measures in all factors. The dependent variable was data fit to the model assessed by the Fisher *Z* transform of Pearson correlation coefficient *r*, to normalize its distribution.

Six methods were used to measure NLC length in all NLCs: three direct methods (axial, end-to-end and polygonal) and three model based methods (using polynomials-2, -3 and -4 as NLC models). Axial length was obtained as the Euclidean distance between the external planes of the two slices delimiting the NLC. End-to-end length was taken as the distance between canal central points on its outer delimiting planes. Polygonal length was obtained as the sum of the lengths of the linear segments joining the 3D centroids and adding their extensions from the two extreme centroids to their respective outer planes delimiting the NLC. The lengths of polynomial curves were computed by numerical methods with a precision greater than 0.1 mm. The effect on length results of the measurement method (axial, end-to-end, polygonal, polynomial-2, polynomial-3 and polynomial-4), controlling for the correlation between canals of the same subject (left and right), was studied with a two factor 6x2 repeated measures ANOVA.

Cross-sectional areas for each slice were determined using five methods: directly on the CT in the image plane (axial method) or in the planes orthogonal to the NLC direction as given by the polygonal and polynomial-2, -3 and -4 models. The mean cross-sectional area represents the average value of the sectional areas of all the NLC segments differentiated in the CT. The independent variable was thus the measurement method, and the dependent variable the mean cross sectional area of each NLC obtained by each method. The five methods were compared by 5x2 factorial design repeated measures ANOVA for both factors: method and laterality (included again to control for correlations among measures in left and right NLC of the same subject).

The minimum axial cross-sectional area represents the area of the smallest section of the NLC. We also compared minimum cross-sectional areas obtained using the five different methods as described above, as well as the distance from the point of minimum area to the orbit end of the NLC.

The NLC variables generated through our proposed segmentation model were: volume (Vol), axial length (AxL), end-to-end length (ExL), polygonal axis length (PolygL), second-order polynomial axis length (Polyn2L), third-order polynomial axis length (Polyn3L), fourth-order polynomial axis length (Polyn4L), mean area of sections normal to the CT axis (SecA), mean area of sections normal to the polygonal axis (SecPolygA), mean area of sections normal to the 2nd order polynomial axis (SecPolyn2A), mean area of the sections normal to the 3rd order polynomial axis (SecPolyn3A), mean area of the sections of the NLC normal to the 4th order polynomial axis (SecPolyn4A), minimum area of sections normal to the TC axis (MinSecA), minimum area of sections normal to the polygonal axis (MinSecPolygA), minimum area of sections normal to the 2nd order polynomial axis (MinSecPolyn2A), minimum area of sections normal to the 3rd order polynomial axis (MinSecPolyn3A), minimum area of sections normal to the 4th order polynomial axis (MinSecPolyn4A), depth of minimum areas of sections normal to the CT axis (MinSecD), depth of minimum areas of sections normal to the polygonal axis (MinSecPolygD), depth of minimum areas of sections normal to the 2nd order polynomial axis (MinSecPolyn2D), depth of minimum areas of sections normal to the 3rd order polynomial axis (MinSecPolyn3D), and depth of minimum areas of sections normal to the 4th order polynomial axis (MinSecPolyn4D).

For each of the 22 variables, mean, standard deviation, median and quartiles were calculated. The normality of data was checked using Kolmogorov-Smirnov and Shapiro-Wilk tests.

Gender differences in all variables were assessed using the Mann-Whitney test.

The image processing programs were developed in Matlab R2014a (MathWorks, Natick, MA, USA) using Image Processing Toolbox, running on Windows 7 Professional 64 bits, installed in a laptop with i5 M540 processor of 2’53 Gh and 4GB of RAM. Data analysis was performed using SPPS 22 (IBM). The DICOM viewer Centricity (GE) was used by a radiologist to display images of diagnostic quality, identify extreme slices of each NLC and also to identify contours for validation of automated segmentation. When randomization was necessary, true random numbers were obtained from atmospheric noise [16].

## Results

Inclusion criteria were met by 18 CT scans of 36 NLC in 18 healthy participants. Median participant age was 43 years (26 to 77, interquartile range 32). 38.9% were male. No age differences depending on gender were detected (Mann-Whitney U = 98; p = 0.071).

**Fig 3:**
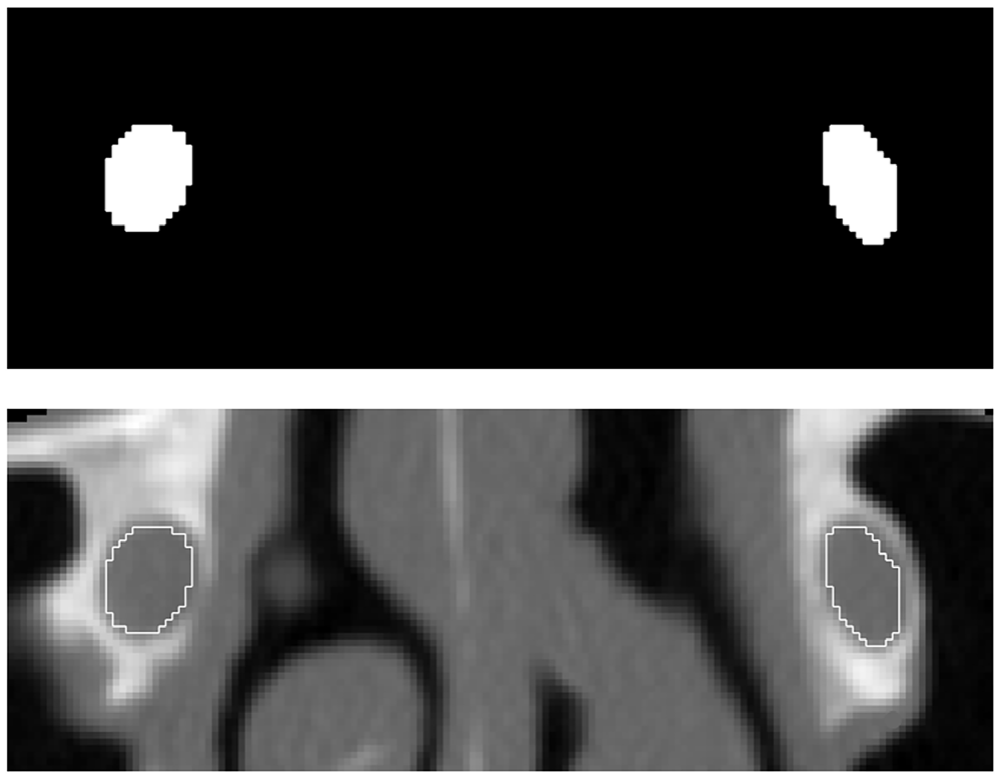
Segmentation results for slice 57 of subject #27 showing above the masks obtained by automatic segmentation for left and right NLC and below the contours of the segmented left and right NLC superimposed to CT slice.

In the 36 NLC, our software was used to segment 591 nasolacrimal regions in 328 different CT slices. Software output was the binary mask for each canal region (Fig 3, above) along with auxiliary images (Fig 3, below) and a set of 54 indices providing information about the shape of each region, including its sectional area and centroid coordinates.

To test the segmentation capacity of the program in terms of robustness and stability segmentation parameters were defined and evaluated on half the NLC samples and then tested on the remaining NCL in the sample. Segmentation was successful in all cases and all measurements could be obtained.

The number of slices available was similar for both canals in a subject (Pearson r = 0.91) yet varied widely between subjects (right NLC mean = 16.39, range 7–21, SD = 4.42; left NLC mean = 16.44, range 6–22, SD = 4.25).

**Fig 4:**
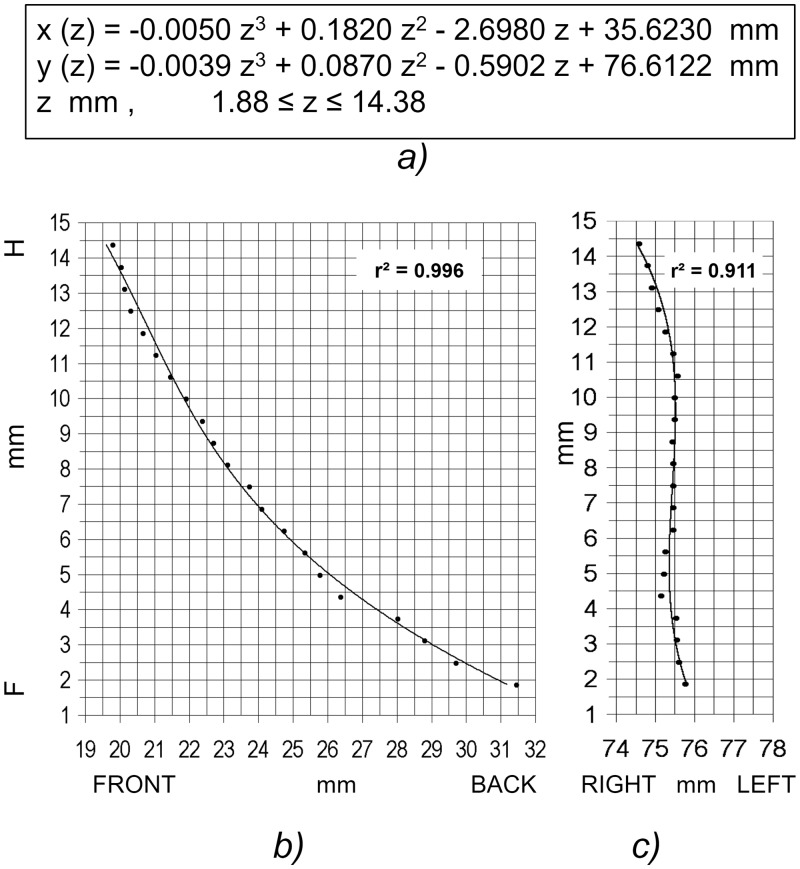
Fit to data of 3rd degree polynomial model (right NLC of subject #20). a) Polynomial model of the NLC axis. b) Sagittal projection. c) Coronal projection.

Mean processing time was around 30 seconds for segmenting each canal. As an example, Fig 4 shows the fit between the polynomial-3 model and the empirical NLC centroids obtained automatically by the program. Goodness of fit was high in both cases (r^2^ = 0.99 and 0.91). The sagittal coordinate plane showed significantly better model fit than the coronal one (F(1, 17) = 23.79; p = 0.000). The order of the polynomial also had a significant effect (Greenhouse-Geisser F(2, 17) = 108.21; p = 0.000), and, as expected the greater the order the better was its fit to the data (p = 0.000 for each pairwise comparison), but at the expense of model parsimony. As a compromise, goodness of fit may be considered sufficient when the lower confidence interval limit (p = 0.05) of the determination coefficient is equal or greater to 0.95. Thus, it may be inferred that the NLC axis will be represented correctly by the vector function in Eq 1 either with cubic polynomials in both components, or with a quadratic polynomial for its sagittal (x) component and a cubic polynomial for its coronal component.

**Fig 5:**
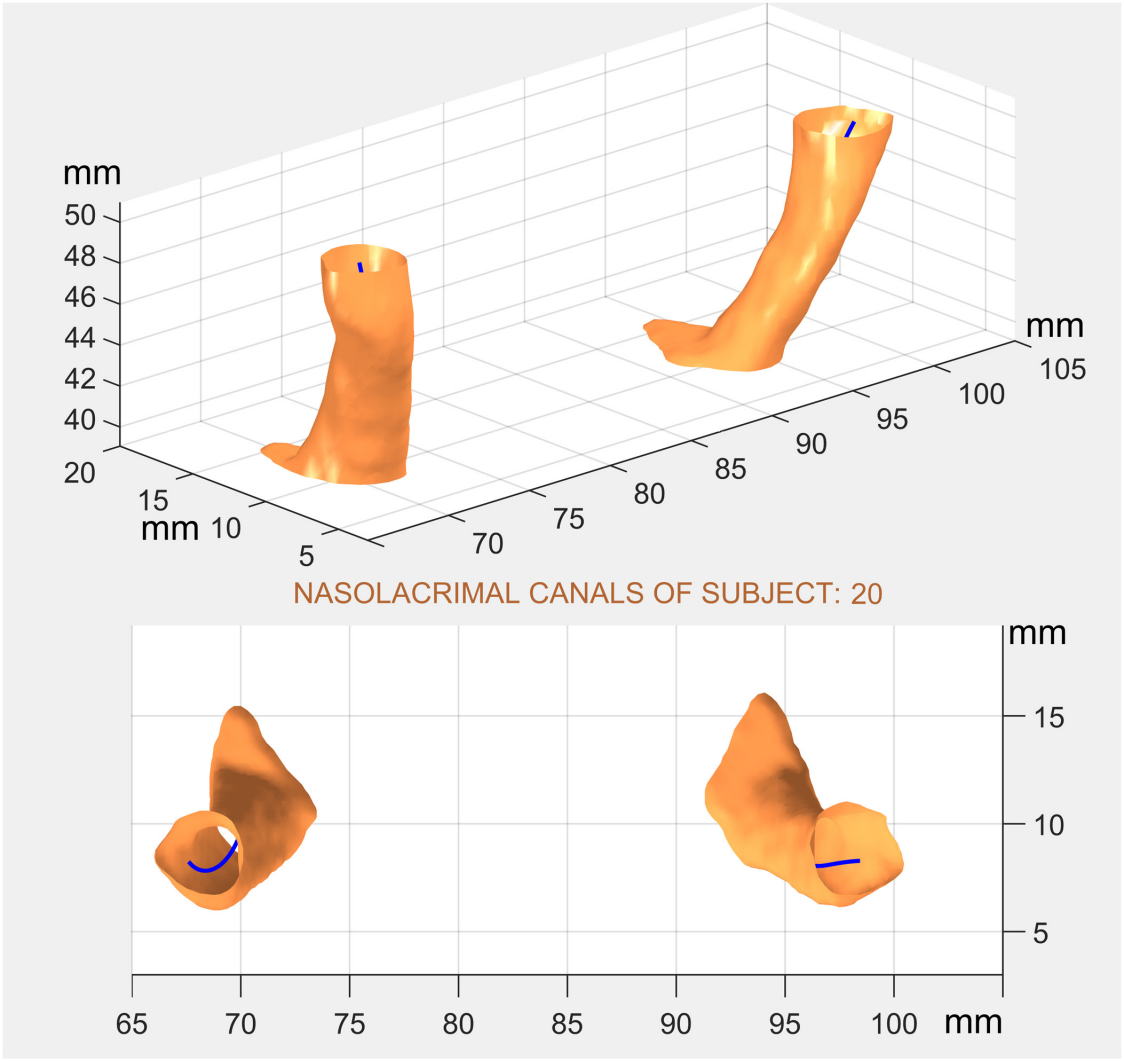
Surface and axis models of NLCs: oblique and overhead views (subject #17).

Polynomial models for the right NLCs of six subjects selected at random are provided in Table 1 as polynomial coefficients for each coordinate, their determination coefficients giving fit to the model, their depth range and the number of slices forming the NLC. The 3-D representation of the model for the NLCs of subject 17 is shown in Fig 5, together with the corresponding axis model.

**Table 1:** Polynomial models for the right NLCs of six subjects selected at random.

Patient	r_x_^2^	a x^3^	+ b x^2^	+ c x	+ d	r_y_^2^	e y^3^	+ f y^2^	+ g y	+ h	z_min_	z_max_	Number of slices
23	0.9975	0.0301	-0.4251	-2.8260	50.8315	0.9979	0.0157	-0.3379	1.5002	66.6736	7.8125	12.1875	7
26	0.9964	-0.0050	0.1820	-2.6980	35.6230	0.9115	-0.0039	0.0870	-0.5902	76.6122	1.5625	14.6875	21
17	0.9964	-0.0036	0.7154	-47.4757	1103.3619	0.9583	0.0016	-0.2846	16.4710	-225.7000	54.6875	67.8125	21
29	0.9967	-0.0047	0.2812	-6.0968	70.5200	0.9130	0.0014	-0.0614	0.8964	73.6686	10.9375	23.4375	20
19	0.9993	0.0001	0.0678	-3.1991	49.9476	0.9602	-0.0008	0.0618	-1.2999	78.7241	10.3125	20.3125	16
13	0.9898	-0.0424	3.5863	-101.5361	1.001.4566	0.9798	-0.0029	0.2722	-8.3017	166.9721	23.4375	30.9375	12

Table 2 provides descriptive data for NLC length measurements.

**Table 2:** Descriptive data for NLC length measurements.

	Mean	Median	Std. Deviation	Percentiles		
				25	50	75
**AxL**	10.26	10.63	2.66	8.13	10.63	12.50
**ExL**	12.84	12.79	2.69	10.12	12.79	14.92
**PoligL**	14.74	14.43	3.04	11.82	14.43	17.01
**Polyn4L**	15.03	14.71	3.07	12.72	14.71	17.39
**Polyn3L**	14.80	14.40	3.07	12.59	14.40	17.01
**Polyn2L**	14.30	14.32	2.82	11.53	14.32	16.53

Correlations between pairs of contralateral NLC lengths ranged from 0.83 to 0.92 using the same method, and from 0.54 to 0.99 among all six methods; all were significant (p = 0.05). According to two factor repeated measures ANOVA, the effect of the method was significant (Greenhouse-Geisser F(1.17, 20.97) = 65.34; p = 0.000) whereas that of laterality was not (F(1, 17) = 2.51; p = 0.13). Pairwise comparisons among methods indicated that the 3rd order polynomial-based method differed significantly from all other methods (p<0.000).

Table 3 provides descriptive data for the NLC sectional area measurements.

**Table 3:** Descriptive data for the NLC sectional area measurements.

	Mean	Median	Std. Deviation	Percentiles		
				25	50	75
**SecA**	21.69	20.20	6.77	16.78	20.20	26.31
**SecPoligA**	15.15	13.98	4.36	12.63	13.98	17.41
**SecPolynA2**	11.77	11.77	2.82	10.48	11.77	14.04
**SecPolynA3**	11.43	11.00	2.93	9.88	11.00	13.67
**SecPolynA4**	11.56	11.00	3.24	9.67	11.00	13.88

Mean sectional areas obtained with the five methods were compared by repeated measures ANOVA according to both method and laterality. Correlations between left and right measurements with the same method were ranging from 0.30 to 0.63 using the same method (two significant with p<0.01; three with p>0.05), and from 0.15 to 0.63 among all methods (only four being significant with p<0.05). Results showed a significant main effect of method (Greenhouse-Geisser F(1, 25, 21.30) = 68.34, p = 0.000) and pairwise comparisons of each method with the polynomial-3 method indicated significant differences for the axial (F(1, 17) = 74.82; p = 0.000), polygonal (F(1, 17) = 47.13; p = 0.000) and polynomial-2 (F(1, 17) = 4.63; p = 0.046) and no significant difference with polynomial-4 (F(1, 17) = 0.42; p = 0.52).

Descriptive data for minimum NLC sectional area are provided in Table 4. Descriptive data for depth of minimum NLC sectional area are provided in Table 5. Correlations between left and right minimum NLC sectional area measurements in each subject range from 0.21 to 0.53 using the same method (being significant, p<0.05, for only two methods), and on the same range among all methods (only five being significant, p<0.05). The main effect of method was significant (Greenhouse-Geisser F(2, 17, 41.68) = 43.52, p = 0.000) and pairwise comparisons with the polynomial-3 method revealed significant differences only for the axial (F(1, 17) = 59.53; p = 0.000), and non significant for polygonal (F(1, 17) = 0.78; p = 0.39) and polynomial-2 (F(1, 17) = 1.00; p = 0.33) and polynomial-4 method (F(1, 17) = 0.15, p = 0.70).

**Table 4:** Descriptive data for minimum NLC sectional area.

	Mean	Median	Std. Deviation	Percentiles		
				25	50	75
**MinSecA**	13.24	12.56	4.53	10.43	12.56	14.39
**MinSecPoligA**	8.69	9.09	3.35	5.95	9.09	10.62
**MinSecPolyn2A**	7.62	7.63	2.64	5.52	7.63	9.46
**MinSecPolyn3A**	7.40	7.57	2.69	5.03	7.57	8.75
**MinSecPolyn4A**	7.19	7.58	2.67	4.77	7.58	8.87

**Table 5:** Descriptive data for depth of minimum NLC sectional area.

	Mean	Median	Std. Deviation	Percentiles		
				25	50	75
**MinSecD**	4.03	4.06	2.83	1.56	4.06	6.25
**MinSecPoligD**	7.85	7.63	3.82	5.44	7.63	11.24
**MinSecPolyn2D**	7.71	7.54	2.32	5.86	7.54	9.64
**MinSecPolyn3D**	8.19	8.31	3.58	5.51	8.31	11.25
**MinSecPolyn4D**	8.08	9.43	3.99	5.39	9.43	11.33

Mean NLC volume was 215.19 mm^3^ (95% CI: 192.04–238.06). Median 213.1 mm^3^, Q_25_ 161.93mm^3^, Q_75_ 265.75 mm^3^.

Significant gender differences were detected for the measurements AxL (U = 57.5; p = 0.003; median difference = 4.375 mm in favour of males) and SecA (U = 66; p = 0.004; median difference = 5.15 mm in favour of females).

An example of the 3D reconstruction of the NLC is provided in Figs 1 and 5. Corresponding virtual reality colour models allowing 3D rotation are available at www.drajanez.com/nlc3Dmodels.

Data underlying the surface model of subject #17 are provided in S1 File; MATLAB programmes to get such model are provided in S2 File. The sequences of the centroids underlying axis models of table 1 are provided in S3 File. Measurements automatically obtained of all our variables are provided in S4 File.

## Discussion

Computer tomography is a widely available imaging system that provides high-resolution images based on tissue density. Epiphora caused by nasolacrimal duct obstruction accounts for approximately 3% of ophthalmologic consultations and this has prompted an interest in NLC anatomy [1–9]. Although a hollow tube is clearly different from the *in vivo* situation of a canal lined with mucosa, small differences in its diameter could influence tear flow and canal obstruction [1–9]. Racial and gender differences in facial skull dimensions and therefore NLC width have been used to explain the different incidence of nasolacrimal duct obstruction [1–9].

Bony NLC anatomy has been explored in cadaveric studies and using techniques such as X-ray imaging or CT [1–9]. However, as far as we are aware, only Estes et al [9] have published a 3D reconstruction of the NLC obtained by computer assisted manual methods but no one has yet been obtained with sufficient quality and by objective methods and without human interaction.

**Fig 6:**
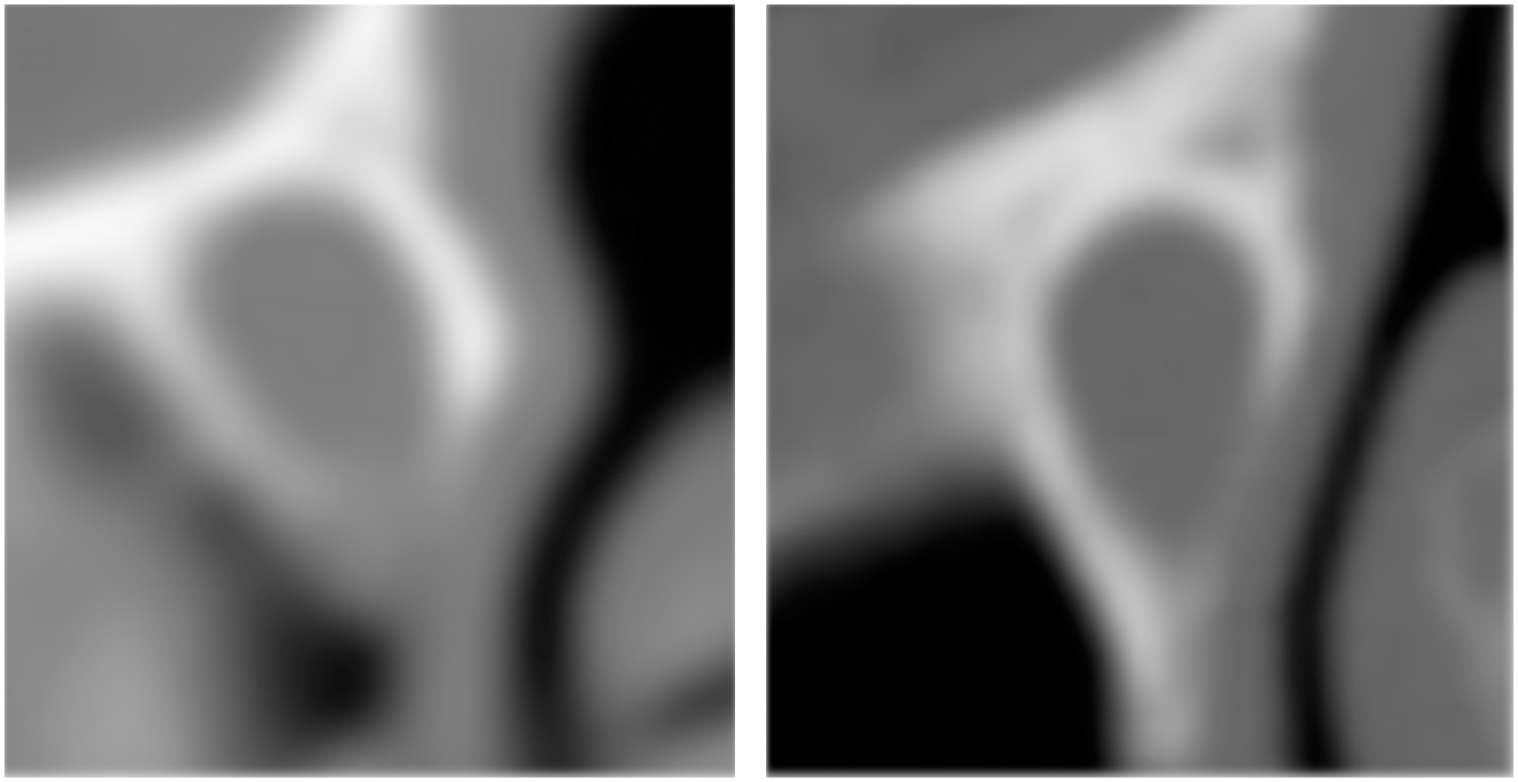
Wall thickness inhomogeneity in the nasolacrimal canals.

Our automatic segmentation method based on *a priori* knowledge and HU values overcomes serious challenges including: a small diameter of the tear ducts; extreme thinness of the canal walls which are sometimes below voxel size Tao [17] and with low HU levels preventing the use of conventional thresholding techniques alone; strong variability in the contrast of canal boundary (Fig 6); a density varying inside the canal, especially when obstructed, often approaching that of thin bones; variable CT procedures and acquisition parameters affecting the geometry of CT volume and its voxels; and random HU fluctuation inherent to CT.

Being automatic it has several benefits over other methods: it fully exploits the information provided by CT by determining sectional area in all slices containing the NLC; it avoids the subjective nature of manual/visual tasks; the real area of the region is measured despite its irregular shape avoiding geometrical approximations; and finally, it is faster than non-automated methods.

When adding the volumes of all voxels identified as comprising the NLC, positive and negative errors in some pixels, possibly arising from a partial volume effect or CT noise, will also be summed. When CT resolution is high, the number of pixels containing this type of random error will also be high, though according to the central limit theorem, positive and negative errors within each NLC will cancel each other out.

The computation of morphometric variables related to the sectional areas and diameters of the NLC is based on automatic determination of the NLC axis. Rather than using the CT axis as reference, four estimates of the NLC axis are provided by our method: polygonal, polynomial-2, polynomial-3 and polynomial-4 to calculate NLC length, sectional area (normal to each axis) and diameter. The morphometric measurements obtained were normally distributed.

The polynomial-3 model showed good fit to the data meaning that its length correctly reflects that of the NLC and may be used as the gold standard for other methods. According to our observations, methods based on the polygonal and polynomial-4 model could be considered equivalent though the polynomial-3 model has two benefits: it is less sensitive than the polygonal model to random deviations in local axis regions due to random errors in HU values; and it is also more parsimonious that the polynomial-4 model.

Our NLC mean lengths obtained with each axis determination method are within the range of: 10 mm reported by Truchot et al [18], 12 mm, Groell et al [19], 12–18 mm, Francisco et al [20], 21.9 mm, Tatlisumak et al [21] and 12.3 mm (men) and 10.8 mm (women), Ramey et al [22].

The normal diameter of the bony NLC has been determined by several authors. Duke-Elder [23] reported a transverse diameter of approximately 4.6 mm based on anatomical observations. Steinkogler [24] measured epoxy resin casts of macerated skulls reporting a transverse diameter of 4.8 mm and anteroposterior diameter of 6.8 mm. Further reported figures include a transverse diameter of 3–5 mm and anteroposterior diameter of 4–8 mm by Cowen and Hurwitz [25]; diameters of 5.6 mm and 5.0 mm respectively in Asian patients measured on CT images by Shigeta et al [2] and a mean minimum transverse diameter of 3.5 mm in 100 healthy adults measured on axial CT images by Janssen et al [26].

In our study, sectional area determinations varied significantly according to the model except when comparing polynomial-3 and -4 indicating a need to replace the conventional axial model with a polynomial axis model, preferably a 3rd order model. Accordingly, NLC sectional area should not be directly determined in axial CT slices because of a strong tendency to overestimate this area. Our new polynomial method for assessing the sectional area of the NLC has various advantages over conventional axial methods: objectiveness, repeatability, automation and exploiting all the information provided by the CT (instead of examining a few selected points). Our model also provides an automatic estimation of the depth at which the minimum sectional area is produced.

Some studies have shown gender differences in the bony NLC. Groessl et al [27] reported that the lower nasolacrimal fossa and middle bony lacrimal duct are significantly smaller in females than males. Janssen et al [26] argued that women had a significantly smaller minimum NLC diameter (0.35 mm on average). Shigeta et al [2] described a significantly smaller bony nasolacrimal canal in female patients; on average anteroposterior diameter was 0.6 mm smaller, transverse diameter 0.3 mm smaller and sectional area 13% smaller. Surprisingly, we only detected gender differences in length and sectional area when computed directly on CT slices, using the conventional axial method.

In conclusion, we here present a new automated segmentation program that provides accurate NLC length, sectional area and volume measurements on CT based on calculating the canal's axis at each measuring point. We consider that this new method could be useful in cases of NLC obstruction, especially in those with repeated treatment failure. Three-dimension models provide a realistic NLC representation that could be used to guide robotic surgery or custom implants production. The fully automated measurement process has gained in objectivity, sensitivity, reproducibility and speed compared to previous methods; consequently its versatility will probably result in making NLC reconstruction easily performable in routine clinical practice whereas the more tedious previous NLC analysis were restricted to the field of research. The method proposed here can be extended to other bony anatomical structures such as intraosseous nerves and vessels, orbit, sinuses, inner ear, internal auditory canal, etc., being therefore useful in other medical specialities. The new methodology can be applied directly to any tomographic study, regardless of manufacturer, CT technology, acquisition (multislice, helical, etc.), reconstruction kernel, voxel size, image resolution, slice spacing and the overlap or gaps between consecutive slices. In future studies, this method can be used to compare the NLC dimensions of subjects with primary acquired NLC obstruction with that of healthy volunteers. Furthermore, we consider it to be a promising tool to determine the association between the dimensions of the NLC and the outcome of any therapeutic intervention performed to treat its pathology.

## Supporting Information

S1 File Data underlying the surface model of subject #17. (ZIP)

S2 File MATLAB programmes to get model of subject #17. (ZIP)

S3 File Voxel dimensions and sequences of the centroids underlying axis models of table 1. (XLSX)

S4 File Demographic variables and measurements automatically obtained for all subjects in our sample. (XLSX)
