# Supplementary figures and images for: Three-Dimensional Reconstruction of the Bony Nasolacrimal Canal by Automated Segmentation of Computed Tomography Images
Source: PLoS One. 2016 May 17;11(5):e0155436. doi: 10.1371/journal.pone.0155436 (PMC4871497; doi:10.1371/journal.pone.0155436)

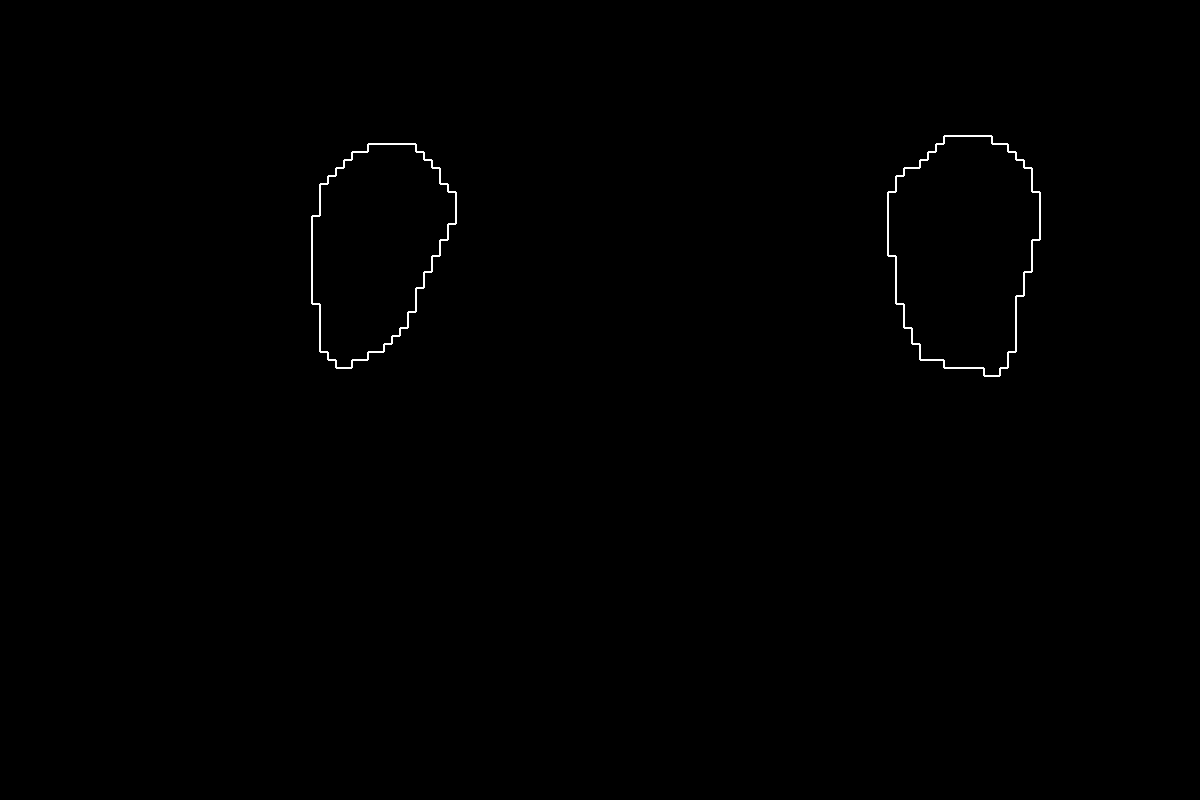

Supplement: S1 File — (ZIP) [file pone.0155436.s001.zip › S1/perfilz_con_p020_ser002_img00064.tif]

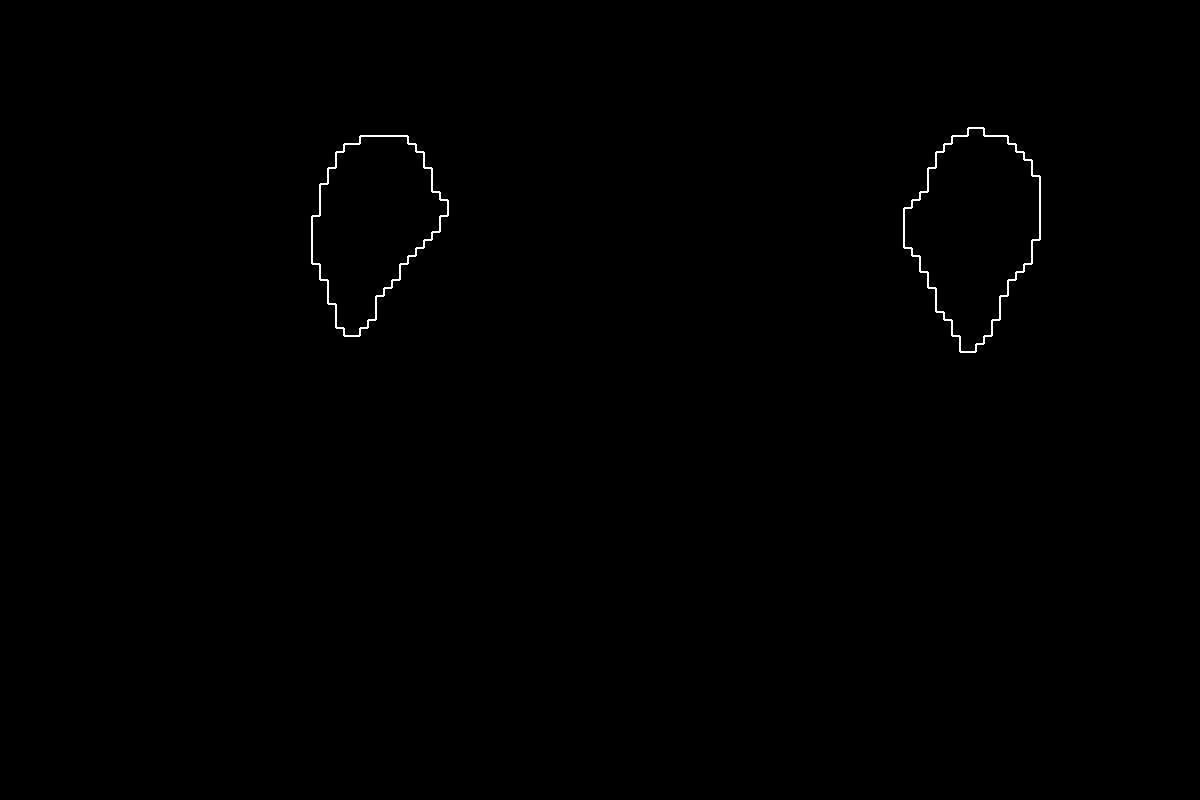

Supplement: S1 File — (ZIP) [file pone.0155436.s001.zip › S1/perfilz_con_p020_ser002_img00065.tif]

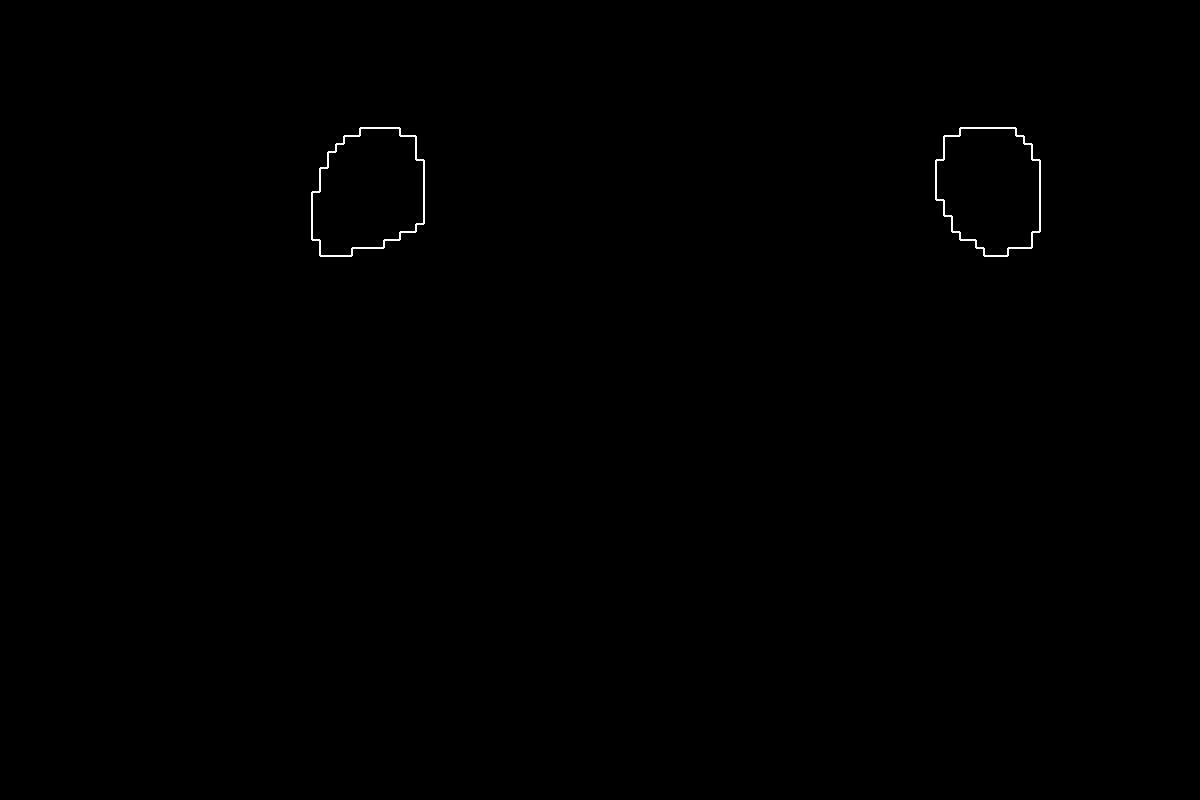

Supplement: S1 File — (ZIP) [file pone.0155436.s001.zip › S1/perfilz_con_p020_ser002_img00066.tif]

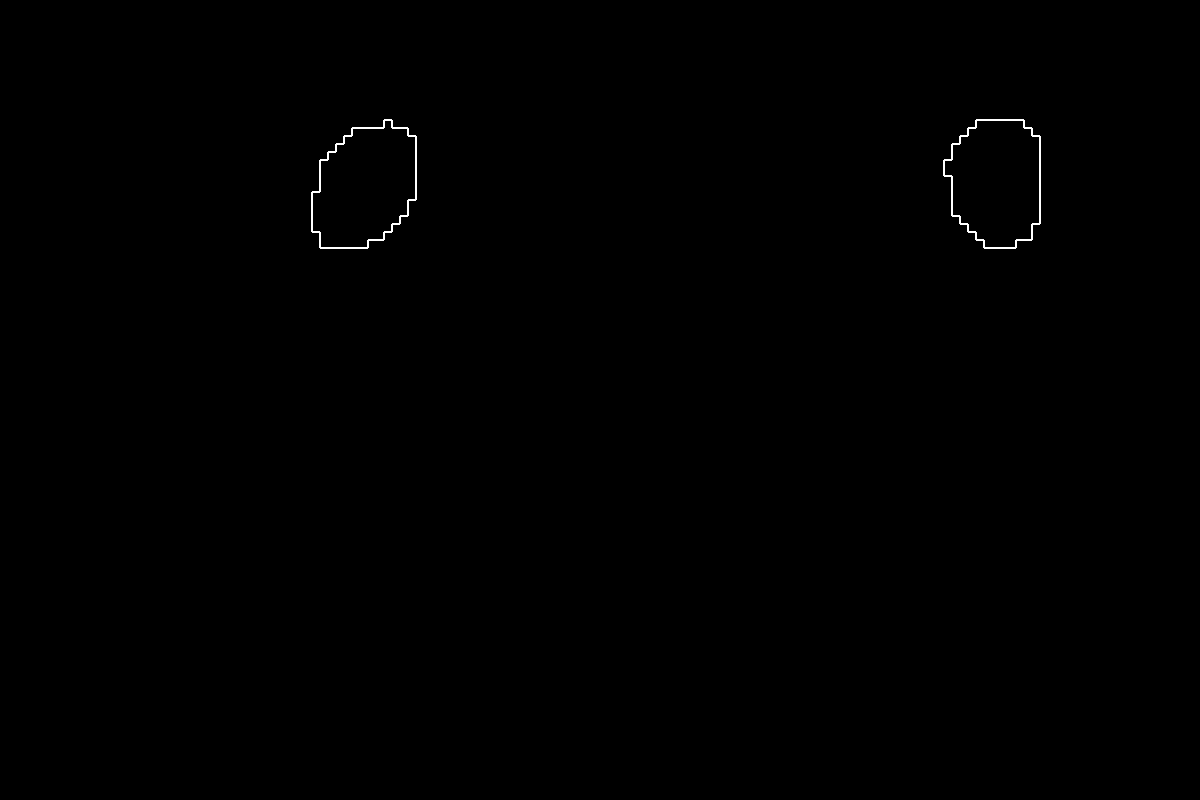

Supplement: S1 File — (ZIP) [file pone.0155436.s001.zip › S1/perfilz_con_p020_ser002_img00067.tif]

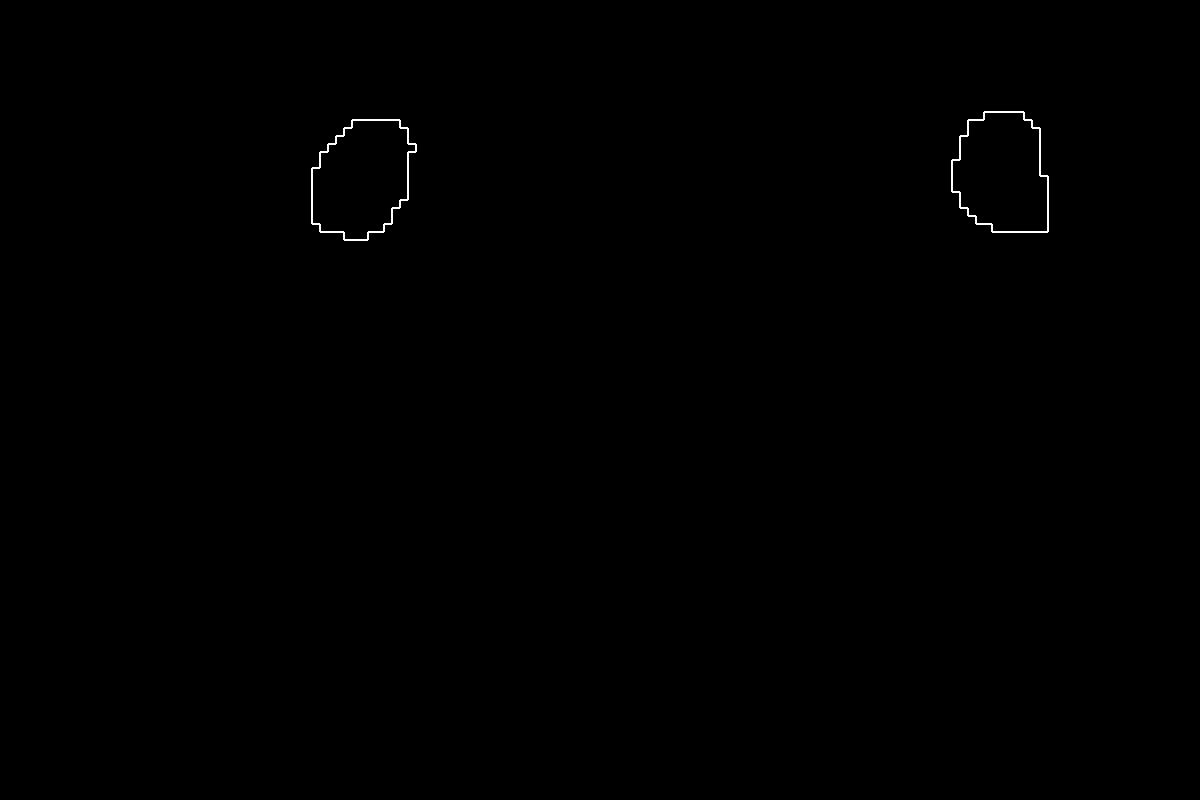

Supplement: S1 File — (ZIP) [file pone.0155436.s001.zip › S1/perfilz_con_p020_ser002_img00068.tif]

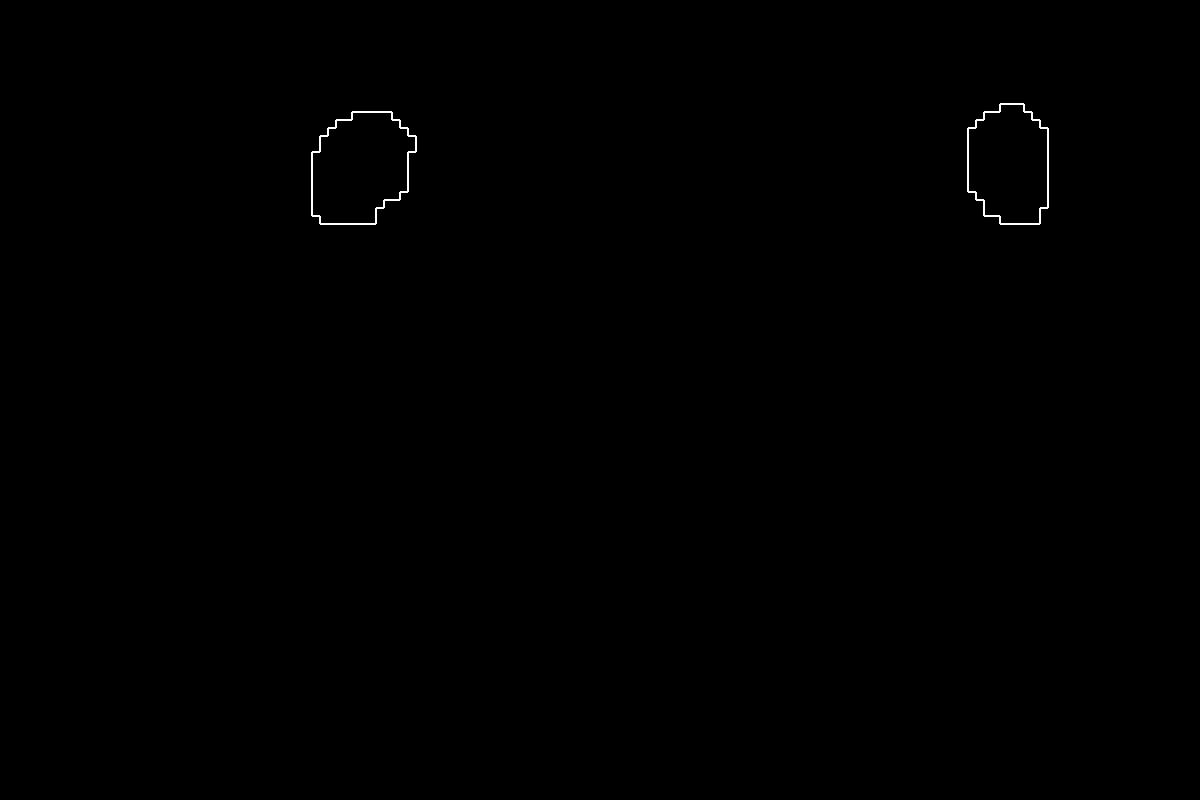

Supplement: S1 File — (ZIP) [file pone.0155436.s001.zip › S1/perfilz_con_p020_ser002_img00069.tif]

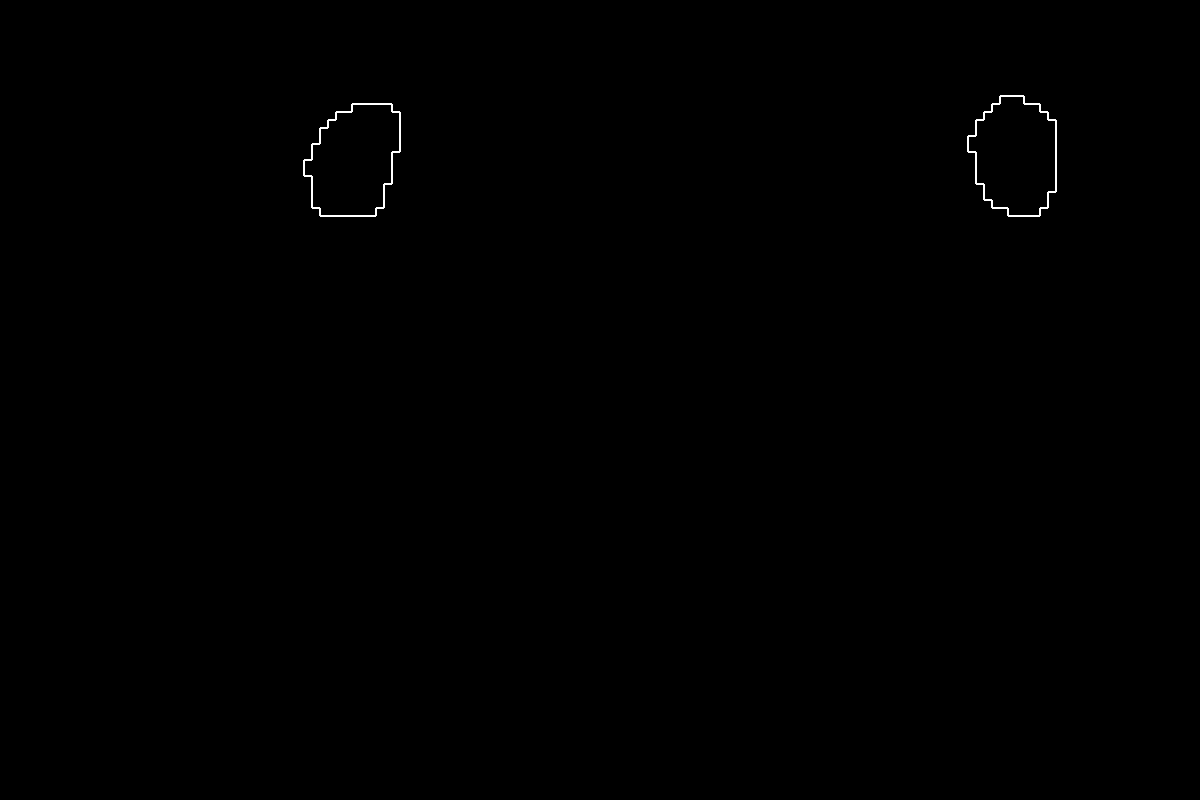

Supplement: S1 File — (ZIP) [file pone.0155436.s001.zip › S1/perfilz_con_p020_ser002_img00070.tif]

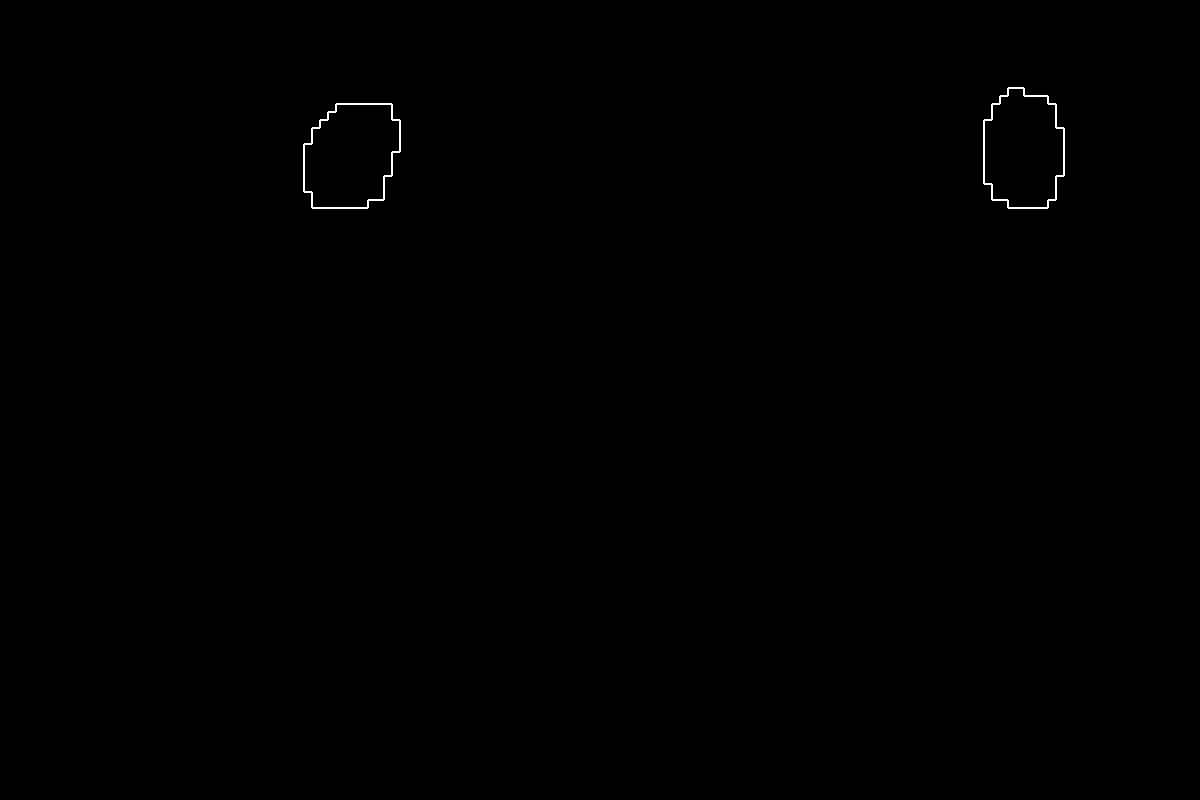

Supplement: S1 File — (ZIP) [file pone.0155436.s001.zip › S1/perfilz_con_p020_ser002_img00071.tif]

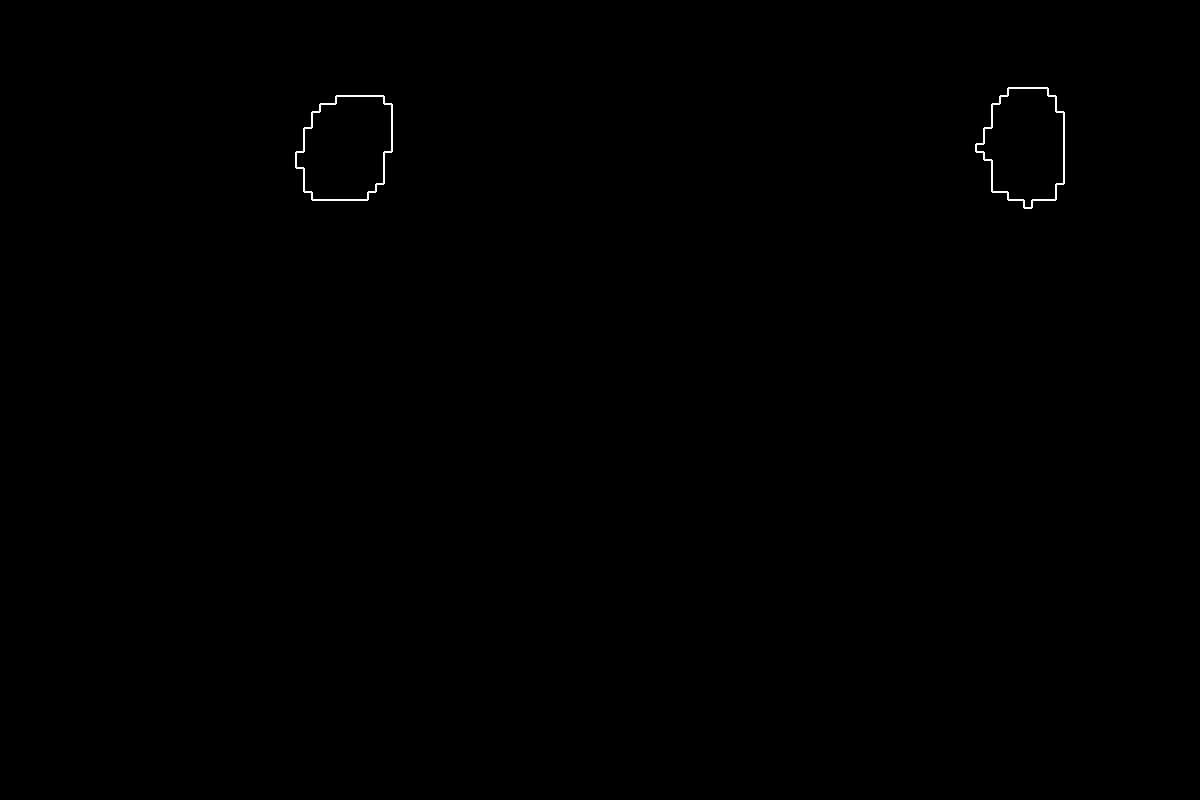

Supplement: S1 File — (ZIP) [file pone.0155436.s001.zip › S1/perfilz_con_p020_ser002_img00072.tif]

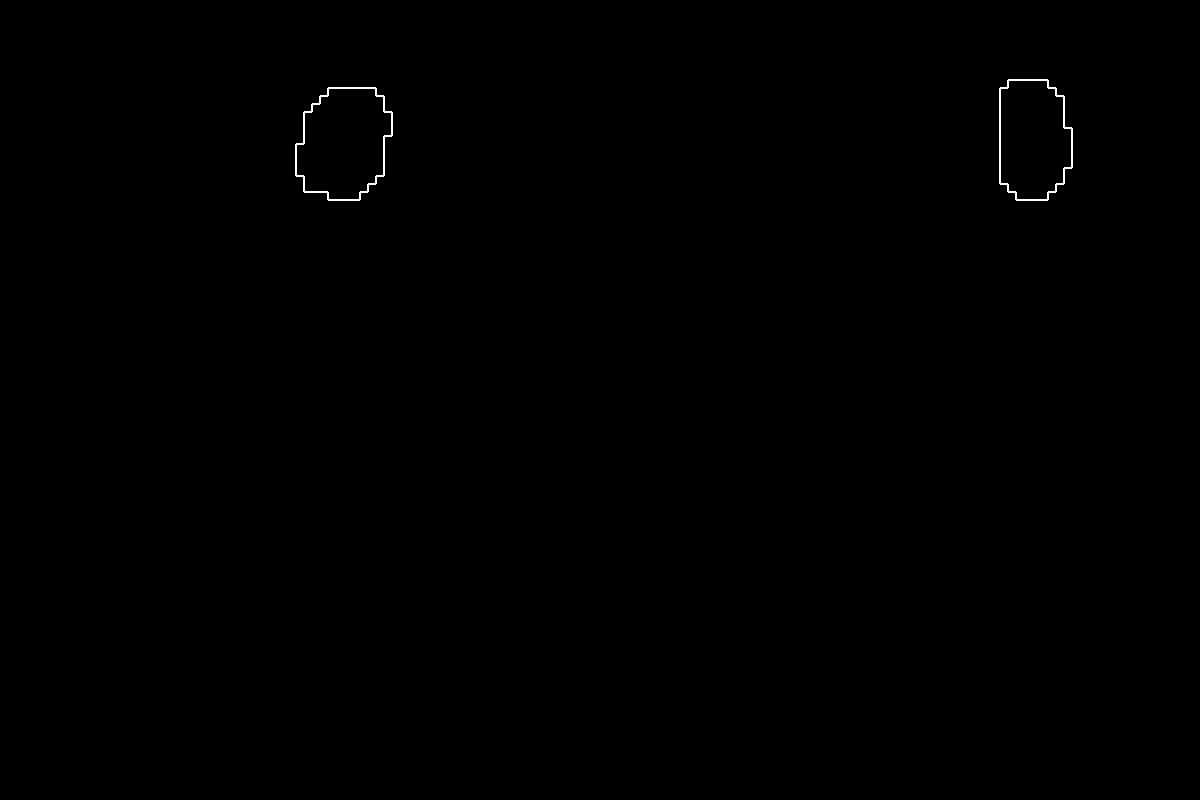

Supplement: S1 File — (ZIP) [file pone.0155436.s001.zip › S1/perfilz_con_p020_ser002_img00073.tif]

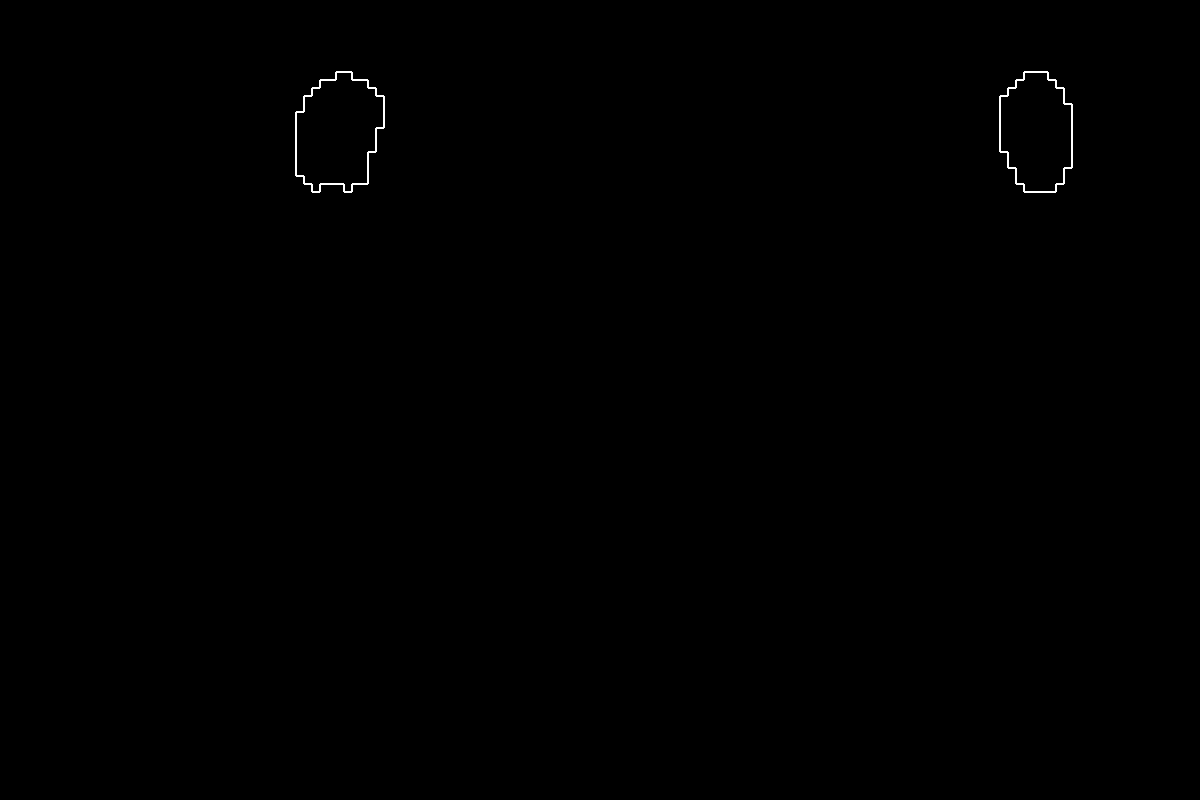

Supplement: S1 File — (ZIP) [file pone.0155436.s001.zip › S1/perfilz_con_p020_ser002_img00074.tif]

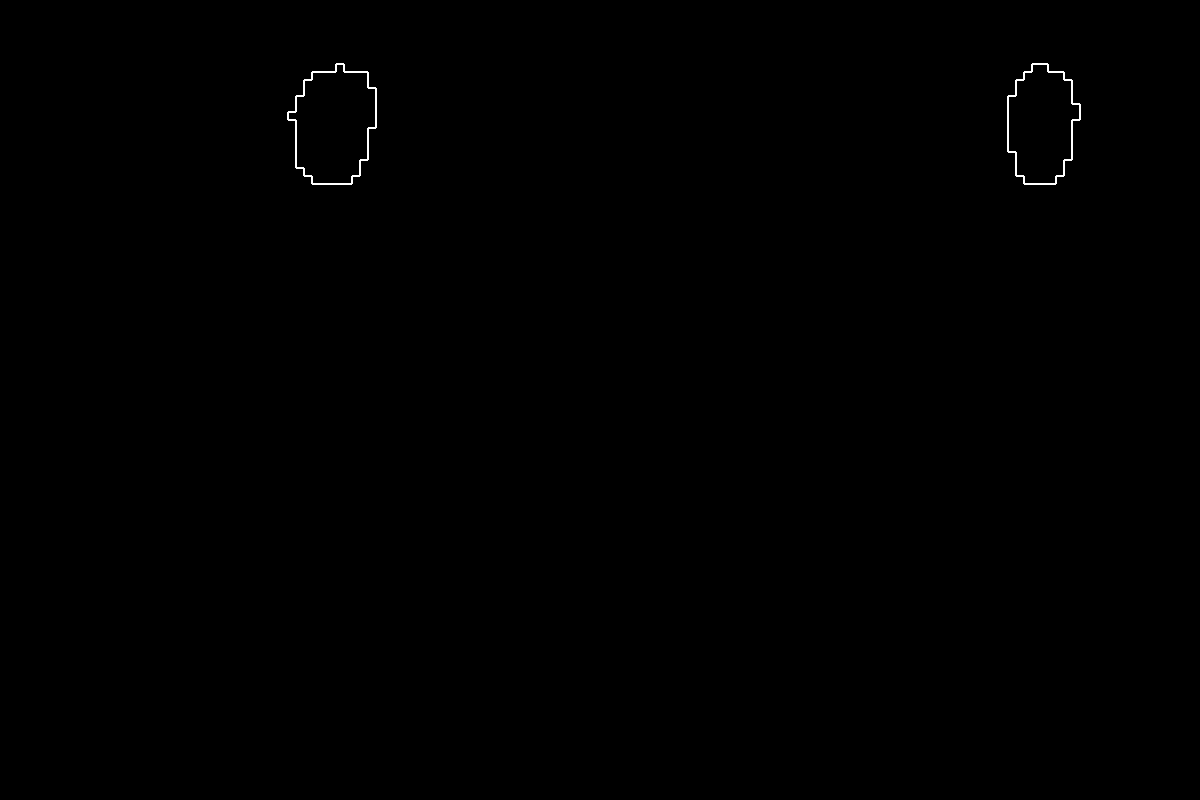

Supplement: S1 File — (ZIP) [file pone.0155436.s001.zip › S1/perfilz_con_p020_ser002_img00075.tif]

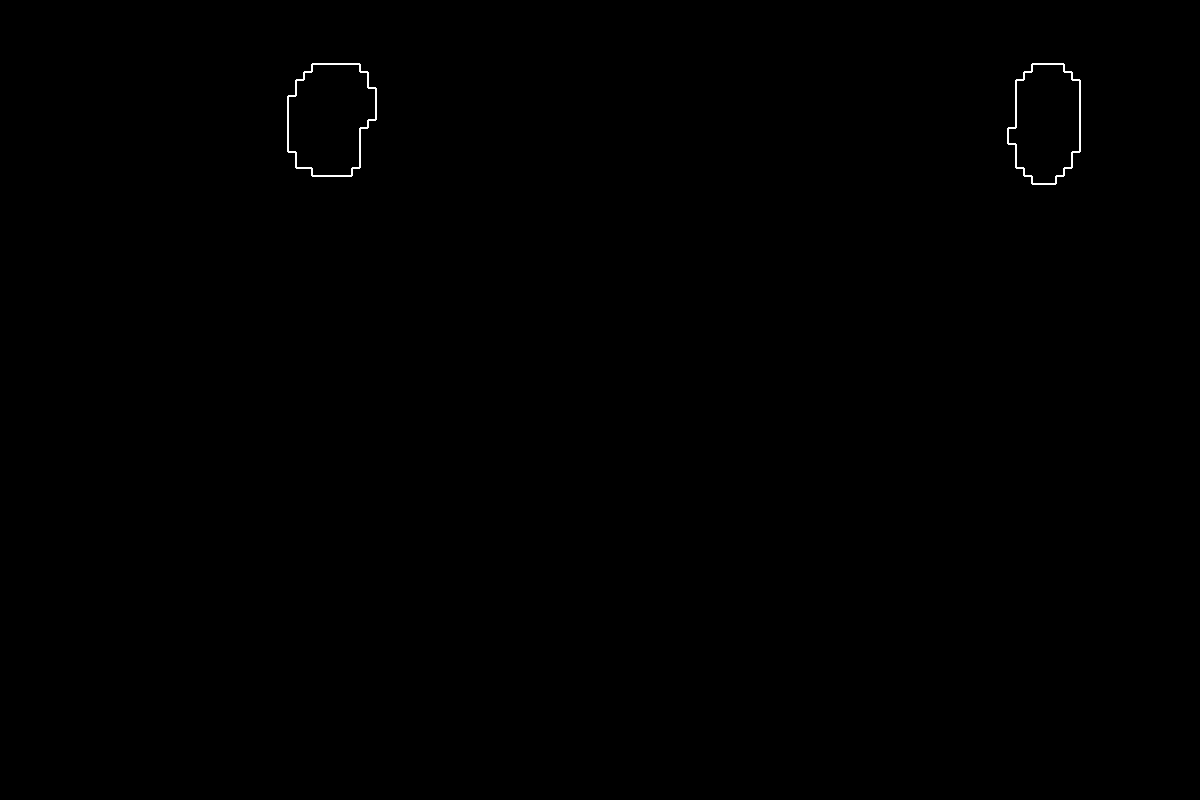

Supplement: S1 File — (ZIP) [file pone.0155436.s001.zip › S1/perfilz_con_p020_ser002_img00076.tif]

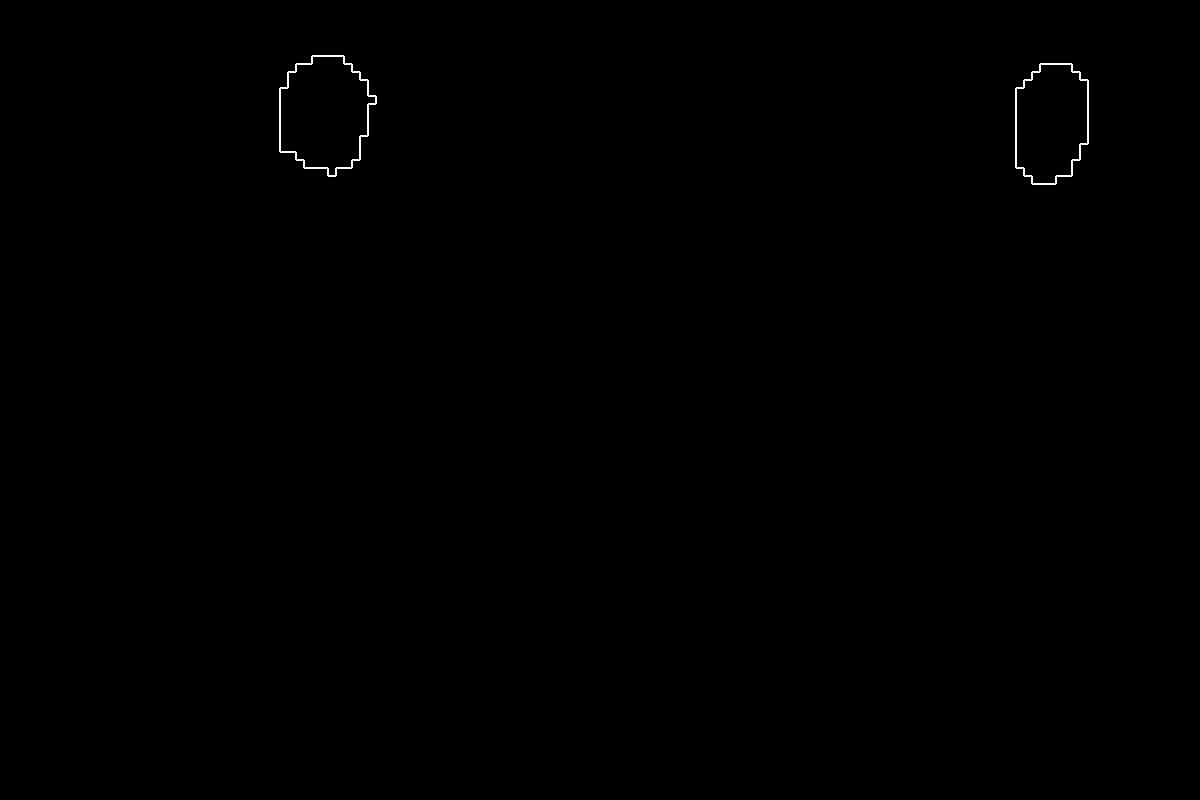

Supplement: S1 File — (ZIP) [file pone.0155436.s001.zip › S1/perfilz_con_p020_ser002_img00077.tif]

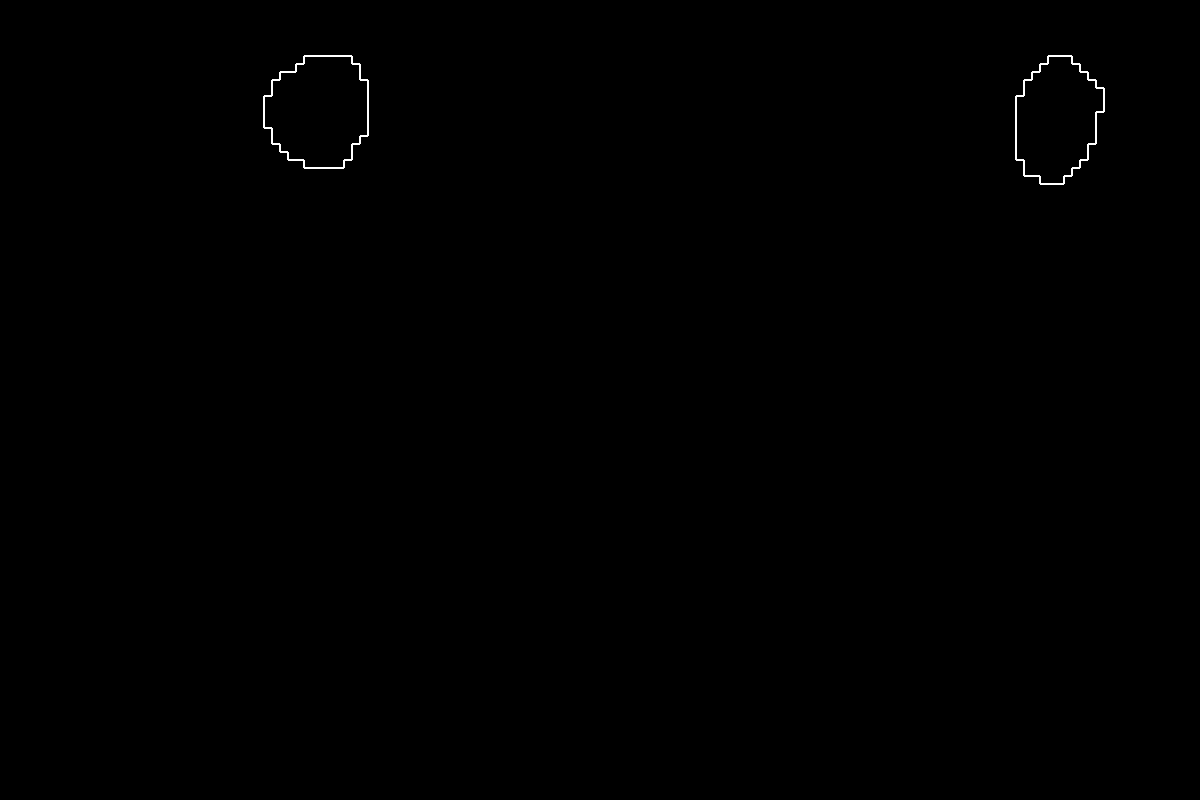

Supplement: S1 File — (ZIP) [file pone.0155436.s001.zip › S1/perfilz_con_p020_ser002_img00078.tif]

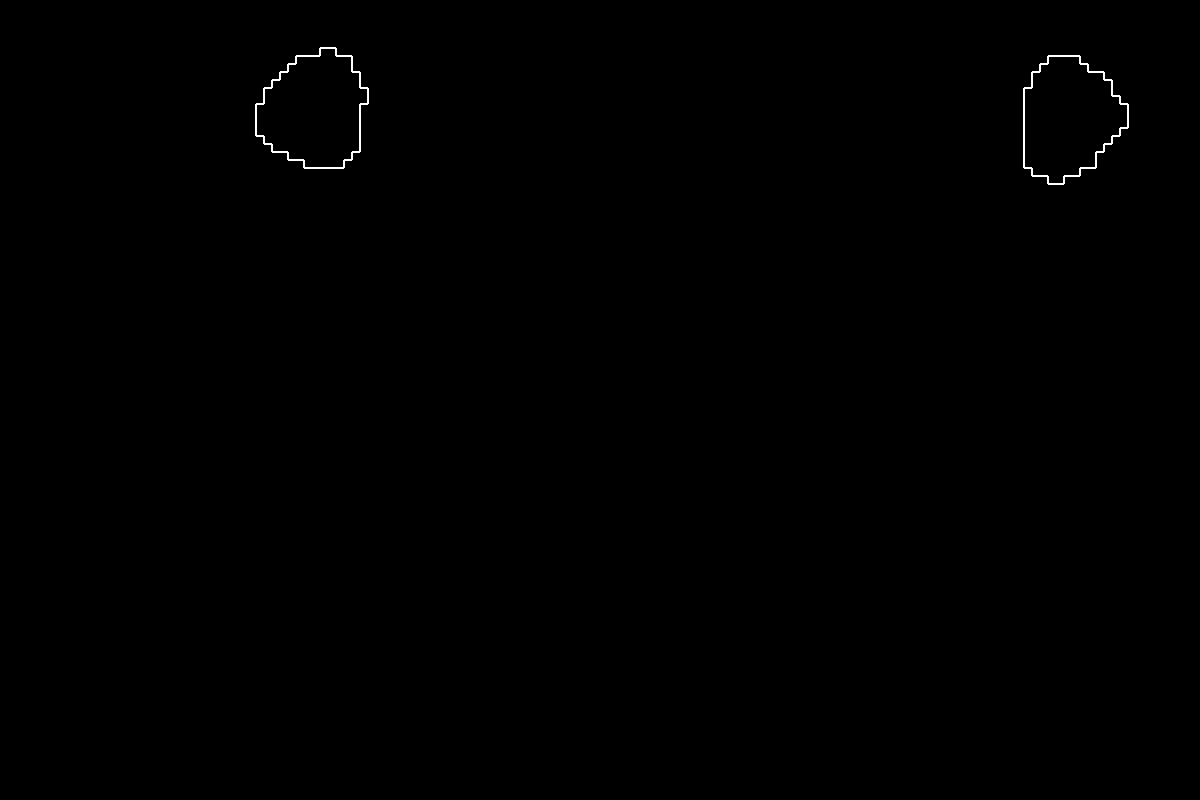

Supplement: S1 File — (ZIP) [file pone.0155436.s001.zip › S1/perfilz_con_p020_ser002_img00079.tif]

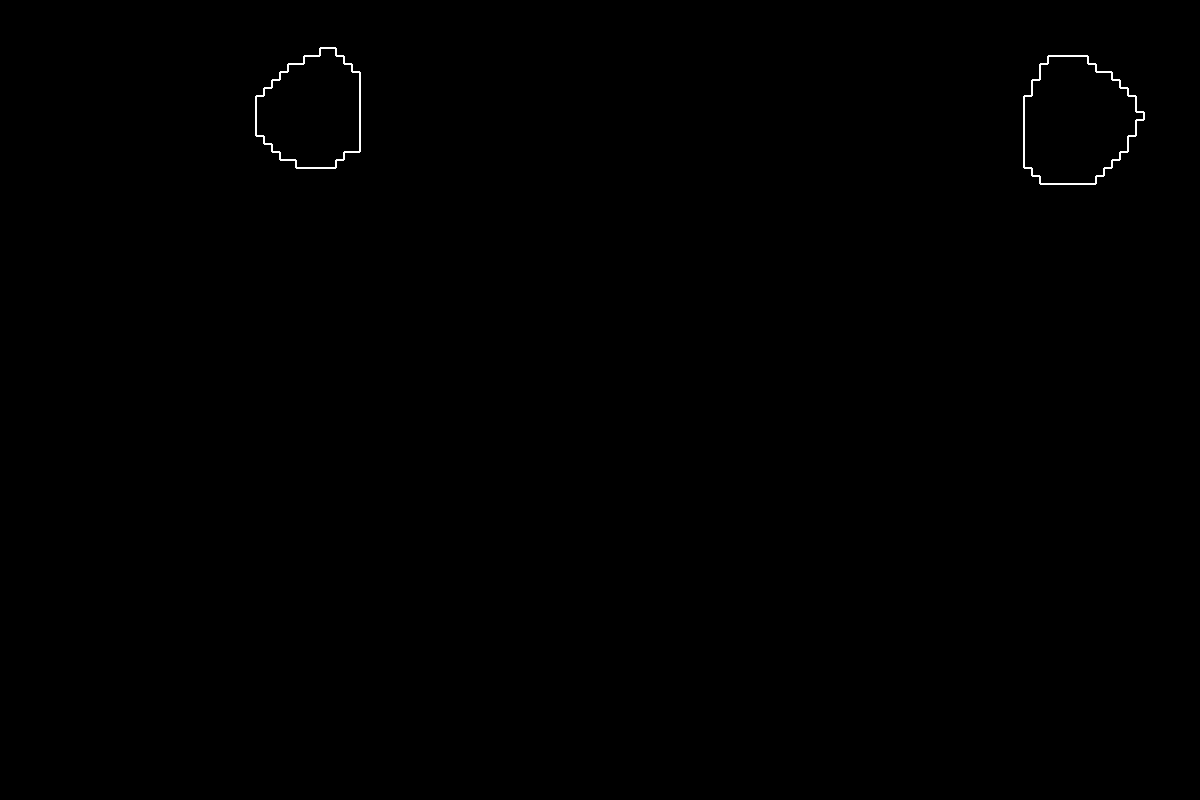

Supplement: S1 File — (ZIP) [file pone.0155436.s001.zip › S1/perfilz_con_p020_ser002_img00080.tif]

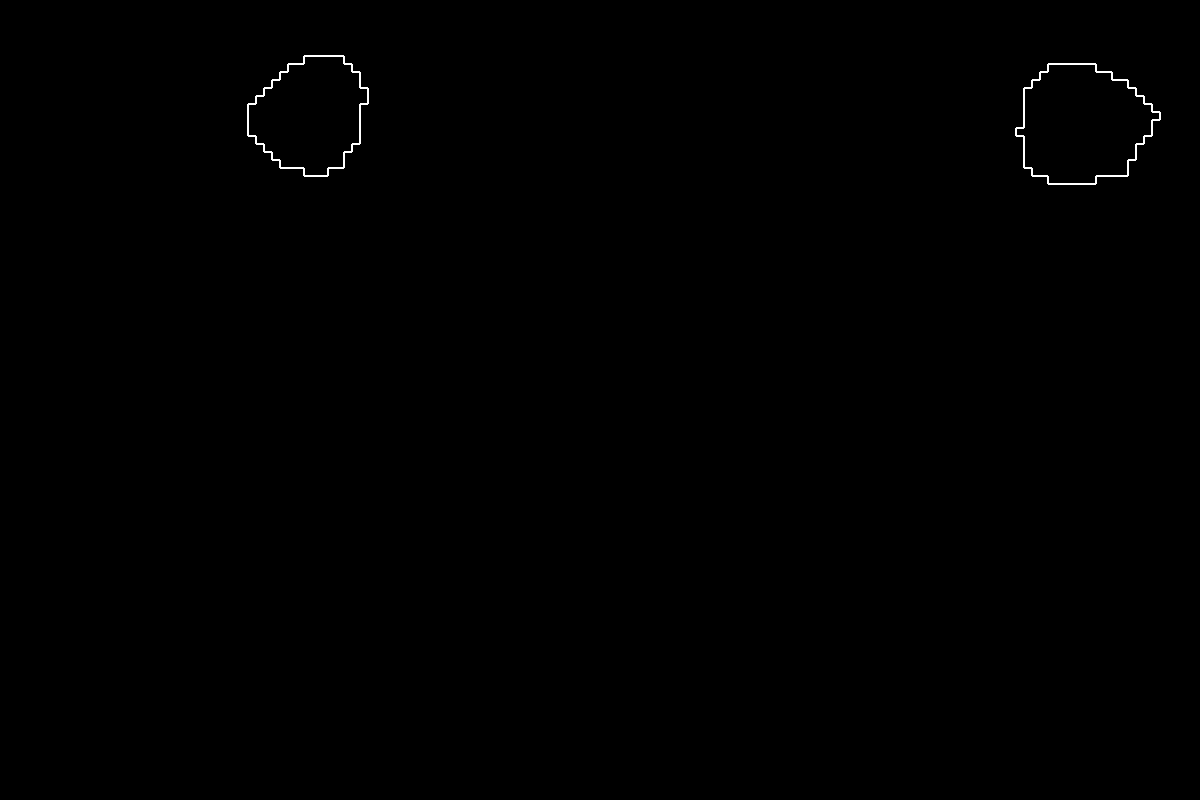

Supplement: S1 File — (ZIP) [file pone.0155436.s001.zip › S1/perfilz_con_p020_ser002_img00081.tif]

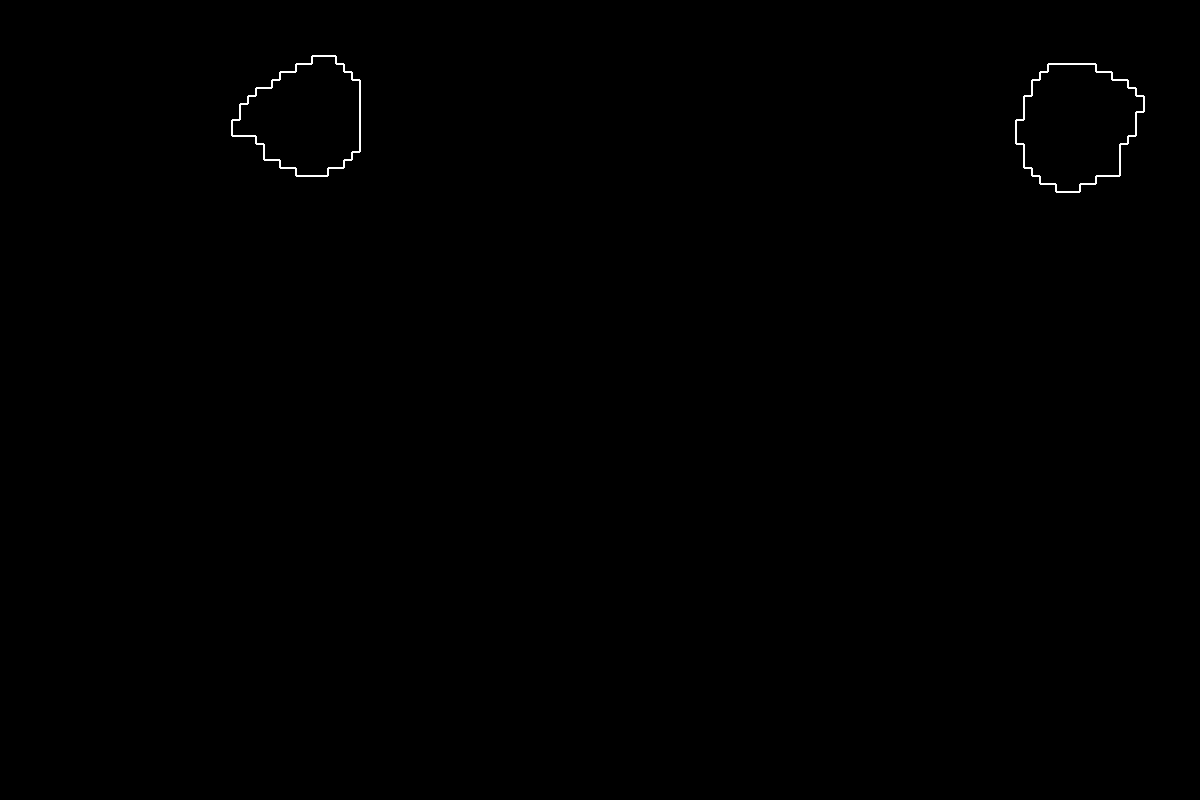

Supplement: S1 File — (ZIP) [file pone.0155436.s001.zip › S1/perfilz_con_p020_ser002_img00082.tif]

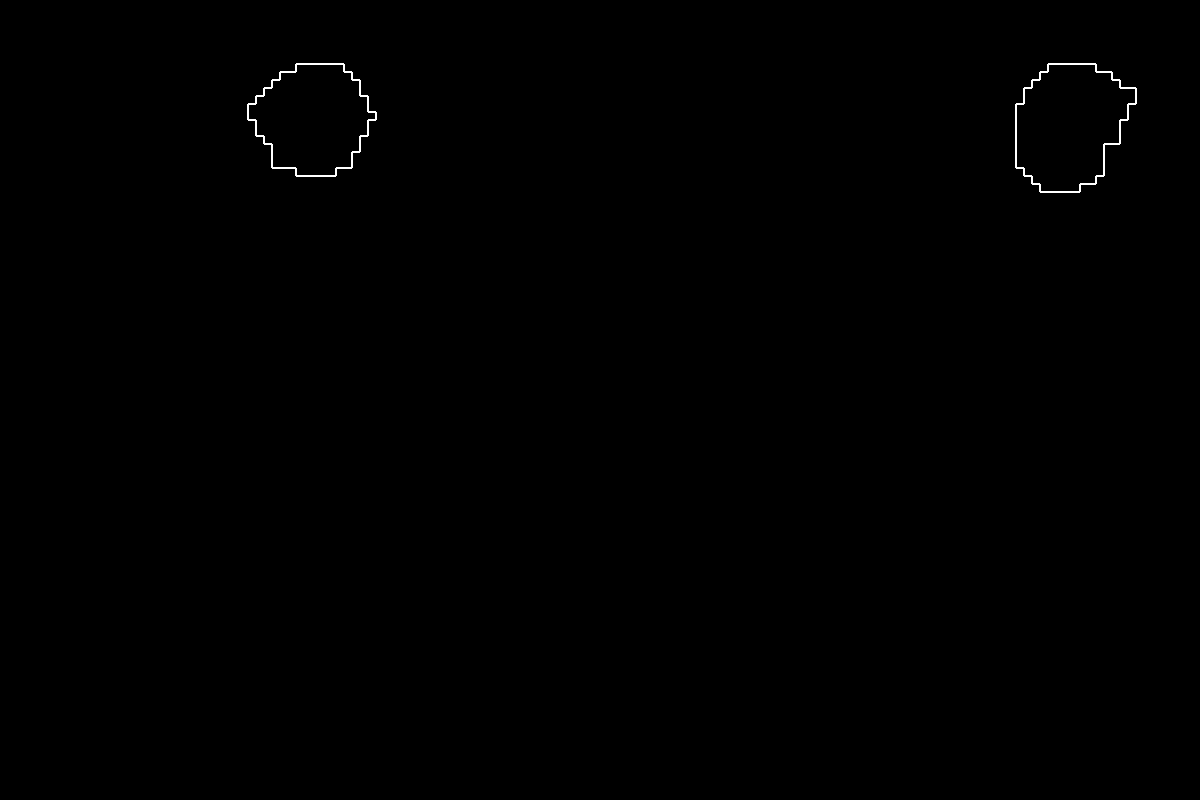

Supplement: S1 File — (ZIP) [file pone.0155436.s001.zip › S1/perfilz_con_p020_ser002_img00083.tif]
